# Alzheimer and Parkinson diseases, frontotemporal lobar degeneration and amyotrophic lateral sclerosis overlapping neuropathology start in the first two decades of life in pollution exposed urbanites and brain ultrafine particulate matter and industrial nanoparticles, including Fe, Ti, Al, V, Ni, Hg, Co, Cu, Zn, Ag, Pt, Ce, La, Pr and W are key players. Metropolitan Mexico City health crisis is in progress

**DOI:** 10.3389/fnhum.2023.1297467

**Published:** 2024-01-12

**Authors:** Lilian Calderón-Garcidueñas, Elijah W. Stommel, Ricardo Torres-Jardón, Jacqueline Hernández-Luna, Mario Aiello-Mora, Angélica González-Maciel, Rafael Reynoso-Robles, Beatriz Pérez-Guillé, Héctor G. Silva-Pereyra, Samuel Tehuacanero-Cuapa, Arturo Rodríguez-Gómez, Ingolf Lachmann, Carolina Galaz-Montoya, Richard L. Doty, Anik Roy, Partha S. Mukherjee

**Affiliations:** ^1^Biomedical Sciences, College of Health, University of Montana, Missoula, MT, United States; ^2^Department of Neurology, Geisel School of Medicine at Dartmouth, Hanover, NH, United States; ^3^Instituto de Ciencias de la Atmósfera y Cambio Climático, Universidad Nacional Autónoma de México, Mexico City, Mexico; ^4^Radiology Department, HMG, Mexico City, Mexico; ^5^Otorrinolaryngology Department, Instituto Nacional de Cardiología, Mexico City, Mexico; ^6^Instituto Nacional de Pediatría, Mexico City, Mexico; ^7^Instituto Potosino de Investigación Científica y Tecnológica AC, San Luis Potosi, Mexico; ^8^Instituto de Física, Universidad Nacional Autónoma de México, Mexico City, Mexico; ^9^Roboscreen GmbH, Leipzig, Germany; ^10^Genetics, GIDP PhD Program, University of Arizona, Phoenix, AZ, United States; ^11^Perelman School of Medicine, Smell and Taste Center, University of Pennsylvania, Philadelphia, PA, United States; ^12^Interdisciplinary Statistical Research Unit, Indian Statistical Institute, Kolkata, India

**Keywords:** air PM_2.5_ pollution, Alzheimer, Metropolitan Mexico City children, quadruple neural proteinopathies, nanoparticles, olfactory bulb, Parkinson, TDP-43

## Abstract

The neuropathological hallmarks of Alzheimer’s disease (AD), Parkinson’s disease (PD), frontotemporal lobar degeneration (FTLD), and amyotrophic lateral sclerosis (ALS) are present in urban children exposed to fine particulate matter (PM_2.5_), combustion and friction ultrafine PM (UFPM), and industrial nanoparticles (NPs). Metropolitan Mexico City (MMC) forensic autopsies strongly suggest that anthropogenic UFPM and industrial NPs reach the brain through the nasal/olfactory, lung, gastrointestinal tract, skin, and placental barriers. Diesel-heavy unregulated vehicles are a key UFPM source for 21.8 million MMC residents. We found that hyperphosphorylated tau, beta amyloid_1-42_, α-synuclein, and TAR DNA-binding protein-43 were associated with NPs in 186 forensic autopsies (mean age 27.45 ± 11.89 years). The neurovascular unit is an early NPs anatomical target, and the first two decades of life are critical: 100% of 57 children aged 14.8 ± 5.2 years had AD pathology; 25 (43.9%) AD+TDP-43; 11 (19.3%) AD + PD + TDP-43; and 2 (3.56%) AD +PD. Fe, Ti, Hg, Ni, Co, Cu, Zn, Cd, Al, Mg, Ag, Ce, La, Pr, W, Ca, Cl, K, Si, S, Na, and C NPs are seen in frontal and temporal lobes, olfactory bulb, caudate, substantia nigra, locus coeruleus, medulla, cerebellum, and/or motor cortical and spinal regions. Endothelial, neuronal, and glial damages are extensive, with NPs in mitochondria, rough endoplasmic reticulum, the Golgi apparatus, and lysosomes. Autophagy, cell and nuclear membrane damage, disruption of nuclear pores and heterochromatin, and cell death are present. Metals associated with abrasion and deterioration of automobile catalysts and electronic waste and rare earth elements, i.e., lanthanum, cerium, and praseodymium, are entering young brains. Exposure to environmental UFPM and industrial NPs in the first two decades of life are prime candidates for initiating the early stages of fatal neurodegenerative diseases. MMC children and young adults—surrogates for children in polluted areas around the world—exhibit early AD, PD, FTLD, and ALS neuropathological hallmarks forecasting serious health, social, economic, academic, and judicial societal detrimental impact. Neurodegeneration prevention should be a public health priority as the problem of human exposure to particle pollution is solvable. We are knowledgeable of the main emission sources and the technological options to control them. What are we waiting for?

## 1 Introduction

Neurodegenerative diseases are usually associated with older people; children are excluded from discussions about Alzheimer’s and Parkinson’s diseases, frontotemporal lobar degeneration (FTLD), or amyotrophic lateral sclerosis (ALS). It is our thesis that this exclusion is seriously flawed.

In 2002, we reported an association of air pollution with the neuropathological hallmarks of Alzheimer’s disease (AD) in dogs living in highly polluted Metropolitan Mexico City (MMC) (Calderón-Garcidueñas et al., 2002). Expression of nuclear neuronal NF-kappa B and iNOS was evident in cortical endothelial cells (ECs) as early as 2 to 4 weeks of age, with subsequent damage to the blood–brain barrier (BBB) and cortical neurons, reflecting diffuse amyloid beta (Aβ_42_) and neurofibrillary tangles (NFT) pathology (Calderón-Garcidueñas et al., 2002). Our statement regarding air pollution and neurodegeneration in 2002 read: *Neurodegenerative disorders such as Alzheimer’s may begin early in life, with air pollutants playing a crucial role* (Calderón-Garcidueñas et al., 2002). A year later (Calderón-Garcidueñas et al., 2003), we documented, in dogs, increased apurinic/apyrimidinic (AP) sites in the olfactory bulb and hippocampus. Nickel (Ni) and vanadium (V) were measured in the olfactory mucosa, olfactory bulb, and frontal cortex using inductively coupled plasma mass spectrometry (ICP-MS). Our results showed that Ni and V were present in a gradient from high concentrations in olfactory mucosa > olfactory bulb > frontal cortex, *de facto* showing the presence of neurotoxic metals in olfactory tissues and their transfer to the frontal lobe.

Documentation of neuroinflammation, i.e., increased expression of cyclooxygenase-2 (COX2) in the olfactory bulb, frontal cortex, and hippocampus and accumulation of Aβ_42_ and alpha-synuclein followed in MMC young adults and children (Calderón-Garcidueñas et al., 2004). We also reported a significant COX2, interleukin-1 beta (IL1β), and CD14—a pattern recognition receptor specific for ligands such as lipopolysaccharide (LPS)—upregulation in the olfactory bulb, frontal cortex, substantia nigra, and vagus nerve, along Aβ42 and α synuclein immunoreactivity in young MMC subjects (Calderón-Garcidueñas et al., 2008b). The presence of nanoparticles (NPs) in olfactory bulb neurons and intraluminal erythrocytes from lungs, frontal lobe, and trigeminal ganglia capillaries, plus the presence of Aβ_42_ plaques in 100% of the apolipoprotein E allele 4, aged MMC 25.1 +/− 1.5y carriers, supported the key role of NPs and APOE4 in the development of Alzheimer’s disease. We noted that “*carriers of the APOE 4 allele could have a higher risk of developing Alzheimer’s disease if they reside in a polluted environment*” (Calderón-Garcidueñas et al., 2008b).

The description of AD pathology in young individuals has also been documented by Braak and collaborators (Braak et al., 2011; Braak and Del Tredeci, 2011). Interestingly, in a German cohort of 2,332 brains of ages 1–100 years, including 32 subjects between ages 1–20 years, the pre-tangle stage a, started in the first decade (Braak et al., 2011), increased progressively with age to stages 1a and 1b and stages I–IV, in a pattern very similar to what we described for MMC subjects (Calderón-Garcidueñas et al., 2018), except for the significantly rapid progression in our highly polluted exposed urbanites.

This study expands the MMC young residents’ development and progression of aberrant protein pathology, including Alzheimer’s disease, Parkinson’s disease, and the spectrum of dysregulation of the 43kD transactive response DNA/RNA-binding protein (TDP-43), the unifying hallmark of frontotemporal lobar degeneration (FTLD) and amyotrophic lateral sclerosis (ALS).

A key focus of the discussion will be to bring to the fore the weakness of the long-maintained dogma of “*the human brain vulnerability to aging”* (Ferrer, 2023), given the documented development of AD, PD, and TDP-43 pathology found in urban children residing in highly polluted environments (Calderón-Garcidueñas et al., 2018). Hence, the detailed documentation of aberrant protein pathology in forensic young people autopsies is critical in light of Del Tredeci and Braak (2022) comment that “*a drawback of postmortem studies generally, namely, a lack of prodromal or very early stage cases.”* We fully endorse this issue. In general, patients with neurodegenerative disease arrive at clinical or pathological examination in late stages, precluding the opportunity to see early neuropathology (Calderón-Garcidueñas et al., 2018).

A larger issue is that multiple neurodegenerative pathologies overlap in elderly people, and they are usually conceptualized as age-related or comorbid neuropathologies (Wakisaka et al., 2003; Braak et al., 2011; Kovacs et al., 2013; Boyle et al., 2018; Nag et al., 2018; Wennberg et al., 2019; Karanth et al., 2020; Robinson et al., 2023). However, we see the same overlap in the first two decades of life in young subjects with no extraneural pathologies, thus implying a common denominator independent of age and comorbidities is at play (Calderón-Garcidueñas et al., 2016, 2017, 2019c, 2022a; Jellinger, 2022).

In this study, we reanalyzed our 186 Metropolitan Mexico City (MMC) forensic cases, with a mean age of 25.7 ± 9.4 years. New antibodies were employed. We provide an updated summary of the light and electron microscopic evaluations of AD, PD, and TDP-43 pathologies. We have expanded our Transmission Electron Microscopy (TEM) analysis and have documented nanoparticles (NPs) through energy-dispersive X-ray spectrometry (EDX).

The overlap of neurodegenerative hallmarks and the significant damage to the neurovascular unit (NVU) starting in childhood has let us to consider the role of nanoparticles in the setting of pollution exposures starting *in utero* (Calderón-Garcidueñas et al., 2022c). The brain electron microscopic features of children and teens are also remarkable, i.e., the breakdown of the NVU being a very early finding that worsens as the infants grow-up in MMC and the presence of NPs damaging endothelial tight-junctions, endothelial and pericytes organelles, and basement membranes. The striking finding of the transfer of NPs from red blood cells (RBCs) to brain endothelium and endothelial RBCs phagocytosis is an important and common observation in MMC residents.

The reader should be aware that the hallmarks of AD, PD, FTLD, and ALS are all present in the first two decades of life in exposed children and teens; these subjects had no extra neural light microscopy pathology and most likely exhibited already cognitive and/or neurological findings at the time of their sudden, unexpected death (Calderón-Garcidueñas et al., 2016, 2017, 2018, 2019c, 2022a,c). The finding of common combustion and friction anthropogenic metals, along with seemingly harmless natural elements and rare industrial metals, makes the issue of NPs of deep interest in neurotoxicology and nano neuropathology and supports a compelling argument to study the emission sources and the related early vulnerability of specific neural cells in common fatal neurodegenerative diseases.

Exposure to polluted environments is associated with the risk of AD, PD, FTLD, and ALS, and as physicians and scientists, we cannot dismiss the lifelong history of intrauterine, indoor and outdoor environmental, occupational, natural disasters, and terrorist act exposures (Jung et al., 2015; Lee et al., 2016; Chen et al., 2017; Alemany et al., 2021; Mortamais et al., 2021; Rhew et al., 2021; Russ et al., 2021; Shi et al., 2021; Zeng et al., 2021; Baranyi et al., 2022; Kritikos et al., 2022; Parra et al., 2022; Rajendran et al., 2022; Shi et al., 2023; Urbano et al., 2023).

## 2 Materials and methods

### 2.1 Study cities and air quality

**Figure 1:**
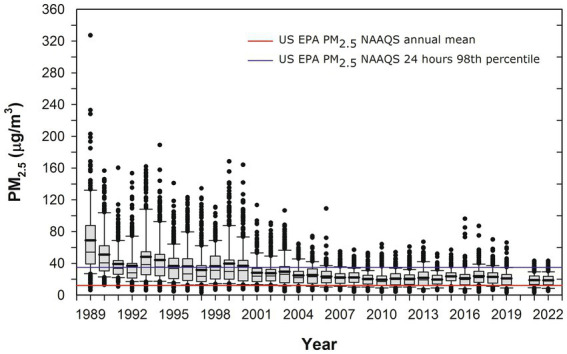
Trend of annual box plots of mean 24-h PM_2.5_ concentrations for five representative MMC monitoring stations from 1989 to 2022 and their comparison with the respective annual and 24-h averages US EPA NAAQS. Box plots from the years before 2004 were estimated from available information on PM_10_ 24-h averages since 1989 and the mean slope of the correlation PM_10_ vs. PM_2.5_ between 2004 and 2007. Data from: http://www.aire.cdmx.gob.mx/aire/default.php.

MMC has 22 million residents who have been chronically exposed to high concentrations of fine particulate matter (PM_2.5_) and ozone O_3_ for the last 3 decades (Dunn et al., 2004; Kleinman et al., 2009; Múgica et al., 2010; Múgica-Alvarez et al., 2012; Kumar et al., 2014; Velasco and Retama, 2017; Molina et al., 2019; Velasco et al., 2019; Caudillo et al., 2020; Zavala et al., 2020; Gustin et al., 2023; USEPA, 2023). In Figure 1, the time series of the annual trend of 24-h PM_2.5_ mean concentrations is shown with data from five representative MMC monitoring stations between 1989 and April 2022 and their comparison with the respective annual and 24-h US EPA NAAQS.

**Figure 2:**
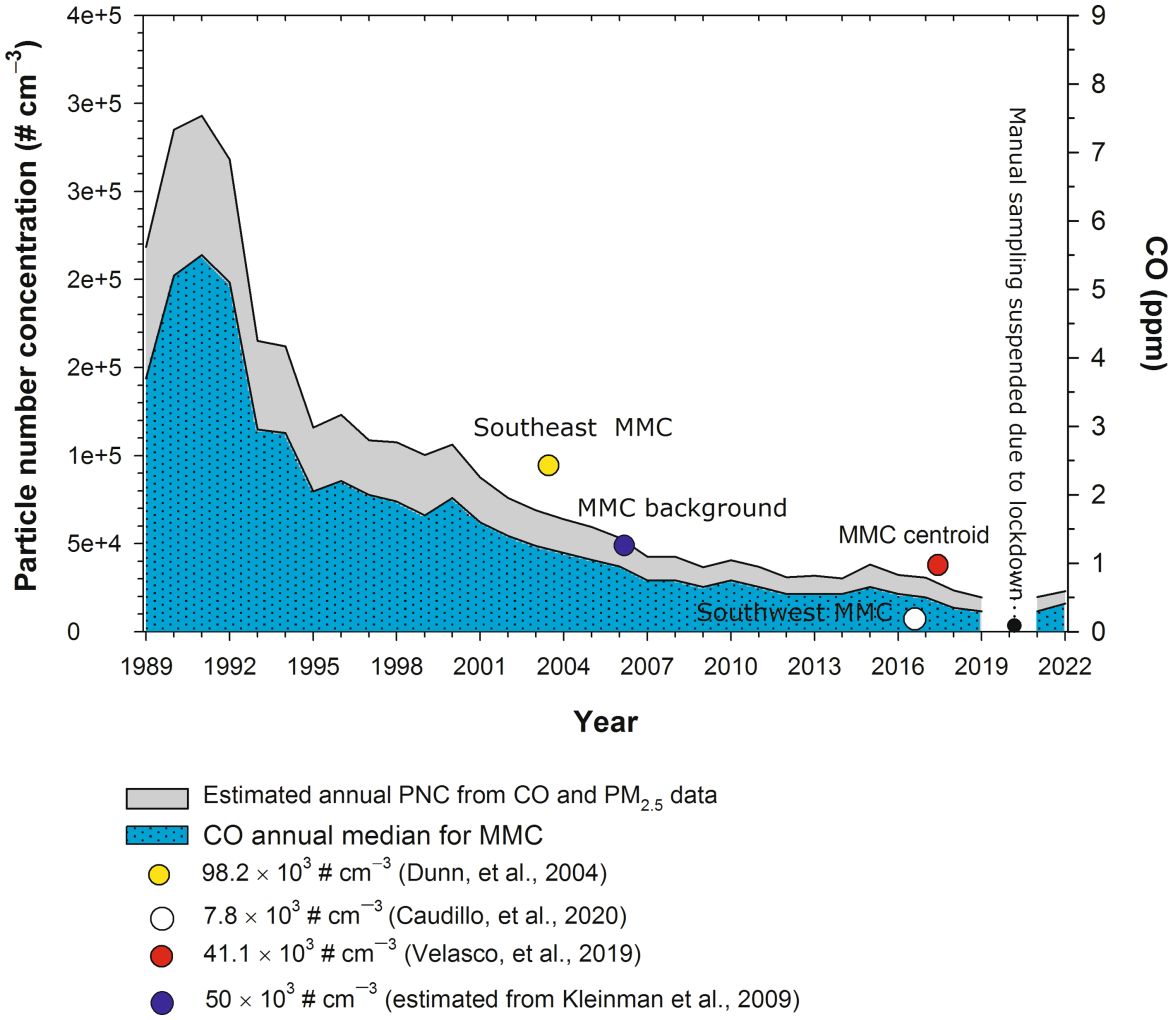
Estimated annual trends of PNC in MMC from 1989 to 2022. PNC values were estimated from the medians of annual measurements of CO and calculated (before 2004) and measured PM_2.5_ (after 2004) in the MMC from 1989 to 2019. The symbols in the figure correspond to the median PNC and the date of measurement reported by Dunn et al. (2004), commercial with median industry and heavy traffic site; the size of measured UFPs between 3 and 15 nm; Caudillo et al. (2020) residential with low traffic site; size of measured UFPs between 20 and 100 nm; Velasco et al. (2019), commercial with moderate to heavy traffic; size of measured UFPs<50 nm; Kleinman et al. (2009), PNC urban background estimated from the extrapolation down to surface of the average UFPs with sizes <100 nm measured by aircraft across the MMC at an altitude average level of 350 m above surface. PM_2.5_ data were obtained at: http://www.aire.cdmx.gob.mx/aire/default.php.

UFPM is defined as particles smaller than 0.1 μm (100 nm) in diameter, commonly referred to as PM_0.1_ a fraction that is not measured in MMC. This PM fraction is measured mostly with specialty instruments by research groups and evaluated as particle number concentration (PNC). To obtain an estimate of the expected PNC historical trend in MMC, we derived an empirical multiple non-linear correlation between available data of simultaneous measurements of NPs, CO, and PM_2.5_ carried out by several research groups in representative places in MMC (Calderón-Garcidueñas et al., 2022a). Figure 2 shows the results.

PNCs were in the range of 300,000 cm^−3^ in the 1990s since CO and PM_2.5_ levels in MMC before 2000 had some of the highest levels of criteria pollutants registered in North America (Molina et al., 2019). It is important to emphasize that MMC residents born before 2002 were exposed to PNCs in the range of 300,000 cm^−3^ from intrauterine life. The PNC trend decreased to the overall average of 44,000 cm^−3^ after 2003, and exposures for MMC residents reached the average PNC for 40 urban areas across Asia, North America, Europe, and Australia after 2003 (Kumar et al., 2014). For this study, we will use UFPM and NPs interchangeably, based on the particle size ≤100 nm.

### 2.2 Study design and samples

The studies involving human participants were reviewed and approved by The University of Montana, Missoula, IRB# 206R-09 and IRB#185-20 for the Protection of Human Subjects in Research. Written informed consent to participate in the clinical study was provided by the participants/participants’ legal guardians. One hundred and eighty-six consecutive autopsies with sudden causes of death not involving the brain were selected for this study. The examination of autopsy materials was approved by the Forensic Institute (20-64/2003) in Mexico City. Subjects aged 27.45 ± 11.89 years were seemingly healthy before their sudden death associated with accidents, homicides, and suicides. Specifically, we ruled out cases with cranial lesions, vascular pathology (including the circle of Willis), evidence of recent or old brain infarcts or head trauma, brainstem abnormalities, and neoplastic pathology. All subjects had unremarkable macro and microscopic examinations of extra-neural key organs. Autopsies were performed 4.1 ± 1.7 h after death between 2004 and 2008, and samples were collected by five trained researchers on weekdays, weekends, and holidays during the 5-year study period. Brains were examined macroscopically, brain sections were selected for light and electron microscopy, and frozen tissues were collected for apolipoprotein E genotyping.

The general characteristics of the study population are seen in Supplementary Table S1. The selected brain sections included a minimum of 12 blocks from the superior and middle frontal cortices (Brodmann Areas 9/46), motor cortex (Brodmann Area 4), miduncal level through medial temporal lobe including hippocampal formation, parahippocampal gyrus, and transentorhinal region, inferior parietal lobule, olfactory bulb, caudate and putamen, thalamus at the level of the lateral hypothalamus, cerebellum, and upper cervical cord. Brainstems were sectioned from the midbrain at the level of the superior colliculi to the lower medulla. Cervical sections C1 and C2 were included in 102 subjects. An average of 10.7 ± 4.18 blocks was obtained from each brainstem and 96 ± 9 slides were examined per block. Paraffin-embedded tissue was sectioned at a thickness of 7 μm and stained with hematoxylin and eosin (HE). Immunohistochemistry (IHC) was performed on serial sections as previously described (Calderón-Garcidueñas et al., 2008b). Antibodies included: β amyloid 17–24, 4G8 (Covance, Emeryville, CA 1: 1500); PHF-tau8 (Innogenetics, Belgium, AT-8 1:1000); Phospho-Alpha-synuclein (Ser129) antibody (PA5-104885) (ThermoFisher); Anti-human α-Synuclein mab 5G4, amino acid sequence 44–57 TKEGVVHGVATVAE, Roboscreen GmbH, Lepzig, Germany, 1:1000; TDP-43 Polyclonal antibody 10782-2-AP, Protein Tech and TDP43 mab2G10, Roboscreen GmbH, Lepzig, Germany both at 1:1000.

Brain tissues were blindly investigated for AD, PD, and TDP-43 pathologies by board-certified neuropathologists and anatomical pathologists. Early subcortical and cortical lesions, the amyloid-β protein phases, brainstem tau pathology, alpha-synuclein, and TDP-43 pathology were the focus of this study (Ward and Gibb, 1990; Jellinger, 1991; Jellinger et al., 2002; Thal et al., 2002; Braak et al., 2003, 2006, 2011, 2017; Alafuzoff et al., 2008; Irwin et al., 2012; Rahimi and Kovacs, 2014; Braak and Del Tredeci, 2015, 2018; Rüb et al., 2016; Del Tredeci and Braak, 2020, 2022). One hundred and twenty-three prefrontal, temporal, and entorhinal cortices, as well as the cerebellum, caudate, frontal Brodmann 4, substantia nigra, locus coeruleus, and/or olfactory bulb blocks, were processed for electron microscopy (EM) (González-Maciel et al., 2017) with a focus on the neurovascular unit and the presence of NPs. Genotyping for APOE allele polymorphisms was done in all cases as previously described (González-Maciel et al., 2017).

The TEM Z-Contrast technique was used to identify NPs in 118 brain samples. The presence of metal, metalloid, and other element NPs was verified through the EDX. Samples were analyzed by scanning electron microscopy with the high-resolution electron microscope SEM7800F, JEOL. The backscattered electron detector was used to observe the metal NPs with a voltage of 15 kV. Chemical analysis measurements were performed with the X-Max^n^ energy-EDX detector from Oxford Instruments. Samples were prepared with an ultra-microtome and placed on gold TEM grids.

### 2.3 Statistical analysis

Our sample size of 186 MMC subjects was defined *a priori* by sampling logistics in the 5-year study period and focused on subjects mostly in the first four decades of life. We concentrated on summary statistics and graphical summary of the targeted staging variables in the first two decades versus the third and fourth and described the two major markers of AD P-tau and amyloid-β_1-42_, α-synuclein as a PD marker and abnormal TDP-43 as a marker for FTLD and ALS. We counted the number of subjects with AD, PD, and TDP-43 pathology within the two cohorts: less than or equal to 20 and equal or more than 21 years. We also calculated the summary statistics of age, gender, and APOE status in each cohort. We constructed Venn diagrams with various intersections of AD, PD, and TDP-43 pathologies in each cohort. We performed the statistical analyses using Excel and the statistical software “R”.^1^

## 3 Results

### 3.1 Air pollution

Metropolitan Mexico City (MMC) is one prime example of uncontrolled urban growth and unsuccessfully controlled environmental pollution (Dunn et al., 2004; Kleinman et al., 2009; Múgica et al., 2010; Múgica-Alvarez et al., 2012; Kumar et al., 2014; Velasco and Retama, 2017; Molina et al., 2019; Velasco et al., 2019; Caudillo et al., 2020; Zavala et al., 2020; USEPA, 2023). Our study included only children and adult residents in MMC. The MMC area is over 2,000 km^2^ and lies at an elevated basin of 2,200 m above sea level. MMC has nearly 22 million inhabitants, over 50,000 industries, and > 5 million vehicles, consuming more than 50 million liters of petroleum fuels per day. In this megacity, MMC motor vehicles release abundant amounts of primary PM_2.5_, elemental carbon, particle-bound polycyclic aromatic hydrocarbons, carbon monoxide, nitrogen oxides, and a wide range of toxins, including lipopolysaccharides, formaldehyde, acetaldehyde, benzene, toluene, and xylenes (Dunn et al., 2004; Kleinman et al., 2009; Múgica et al., 2010; Múgica-Alvarez et al., 2012; Kumar et al., 2014; Velasco and Retama, 2017; Molina et al., 2019; Velasco et al., 2019; Caudillo et al., 2020; Zavala et al., 2020; USEPA, 2023). The high altitude and tropical climate facilitate the reception of strong solar radiation, which enhances ozone production all year (Velasco and Retama, 2017; Zavala et al., 2020) and the formation of secondary particulate matter.

The MMC children of this study were residents in the northern-industrialized and southern-residential zones. Southern Mexico City children have been exposed to significant concentrations of ozone, secondary tracers (NO_3_ˉ), and particles with lipopolysaccharides (PM-LPS), while northern children have been exposed to higher concentrations of volatile organic compounds (VOCs), PM_2.5_, and its constituents: organic and elemental carbon including polycyclic aromatic hydrocarbons, secondary inorganic aerosols (SO_4_^2−^, NO_3_^−^, and NH_4_^+^), and metals (zinc, copper, lead, titanium, manganese, nickel, chromium, and vanadium) (Dunn et al., 2004; Kleinman et al., 2009; Múgica et al., 2010; Múgica-Alvarez et al., 2012; Kumar et al., 2014; Velasco and Retama, 2017; Molina et al., 2019; Velasco et al., 2019; Caudillo et al., 2020; Zavala et al., 2020; USEPA, 2023). Across MMC, residents are exposed to toxic VOCs and polycyclic aromatic hydrocarbons (PAHs), which are complex mixtures containing over 100 compounds associated with fine particles (Múgica et al., 2010; Zavala et al., 2020). These PAHs are abundant in indoor and outdoor air, busy roadways, associated with frying oils and snacks, and a wide range of occupational exposures (Múgica et al., 2010). Historically, MMC subjects in this study have been exposed to significant concentrations of PM_2.5_ above the US EPA standards and ultrafine PM and nanoparticles (NPs) (Dunn et al., 2004; Kleinman et al., 2009; Múgica et al., 2010; Múgica-Alvarez et al., 2012; Kumar et al., 2014; Velasco et al., 2019; Caudillo et al., 2020).

### 3.2 Neuropathology

**Figure 3:**
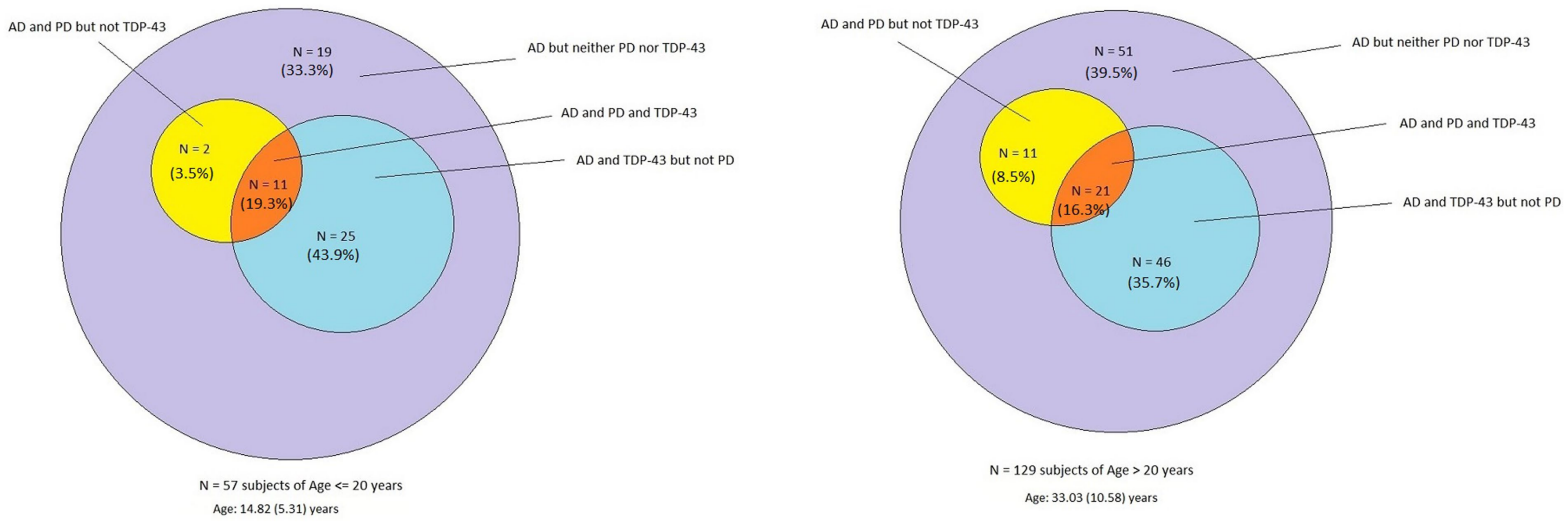
Venn diagram showing the overlap of AD, PD, and TDP-43 pathology in forensic autopsies from 57 children and young adults aged 14.8 ± 5.3 years compared to the 129 subjects with an average age of 33.0 ± 10.5 years. There is no statistical significance in the pathology between cohorts, emphasizing early neuropathology starts in the first two decades of life.

Supplementary Table S1 shows the cases with immunoreactivity (IR) to P-tau, amyloid-β, α-Syn, and TDP43, and in Figure 3, the distribution of P-tau and Aβ_42_ (AD), α-Syn (PD) and TDP43 IR for decades 1 and 2 versus 3 and 4 and their average age is shown.

Remarkably, all 186 cases exhibited AD hallmarks, and the percentage of APOE4 carriers was not statistically different, 12.2 and 13.1% for ≤20-year-old subjects versus ≥21-year-old subjects, respectively (Table 1). There was no statistical significance in the pathology between cohorts, emphasizing their early start in the first two decades of life. Overall, in the 186 cases, the average age of neuropathological AD was 27.4 years, TDP-43 pathology 27.6 years, and PD 29.6 years.

**Table 1:** The age (mean ± SD) of MMC subjects in each diagnosis category, sex, and APOE4 status.

	#	%	Age	Sex	APOE 4#
Subjects ≤ 20y, *n*:57					
AD (≤20)	57	100	14.82 (5.31)	48M, 9F	7
TDP (≤20)	36	63.15	15.21 (5.10)	30M, 6F	5
Non-TDP (≤20)	21	36.8	14.16 (5.73)	18M, 3F	2
PD (Age ≤ 20)	13	22.8	15.81 (5.45)	9M, 4F	2
Non-PD (Age ≤ 20)	44	77.1	14.53 (5.29)	39M, 5F	5
Subjects ≥ 21y, *n*:129					
AD (≥21y)	129	100	33.03 (10.58)	114M, 15F	17
TDP (≥21y)	67	51.9	34.26 (11.84)	52M, 15F	9
Non-TDP (≥21y)	62	48.0	32.02 (9.40)	62M, 0F	8
PD (≥21y)	32	24.8	35.31 (14.40)	26M, 6F	5
Non-PD (≥21y)	97	75.1	32.28 (8.95)	88M, 9F	12

**Figure 4:**
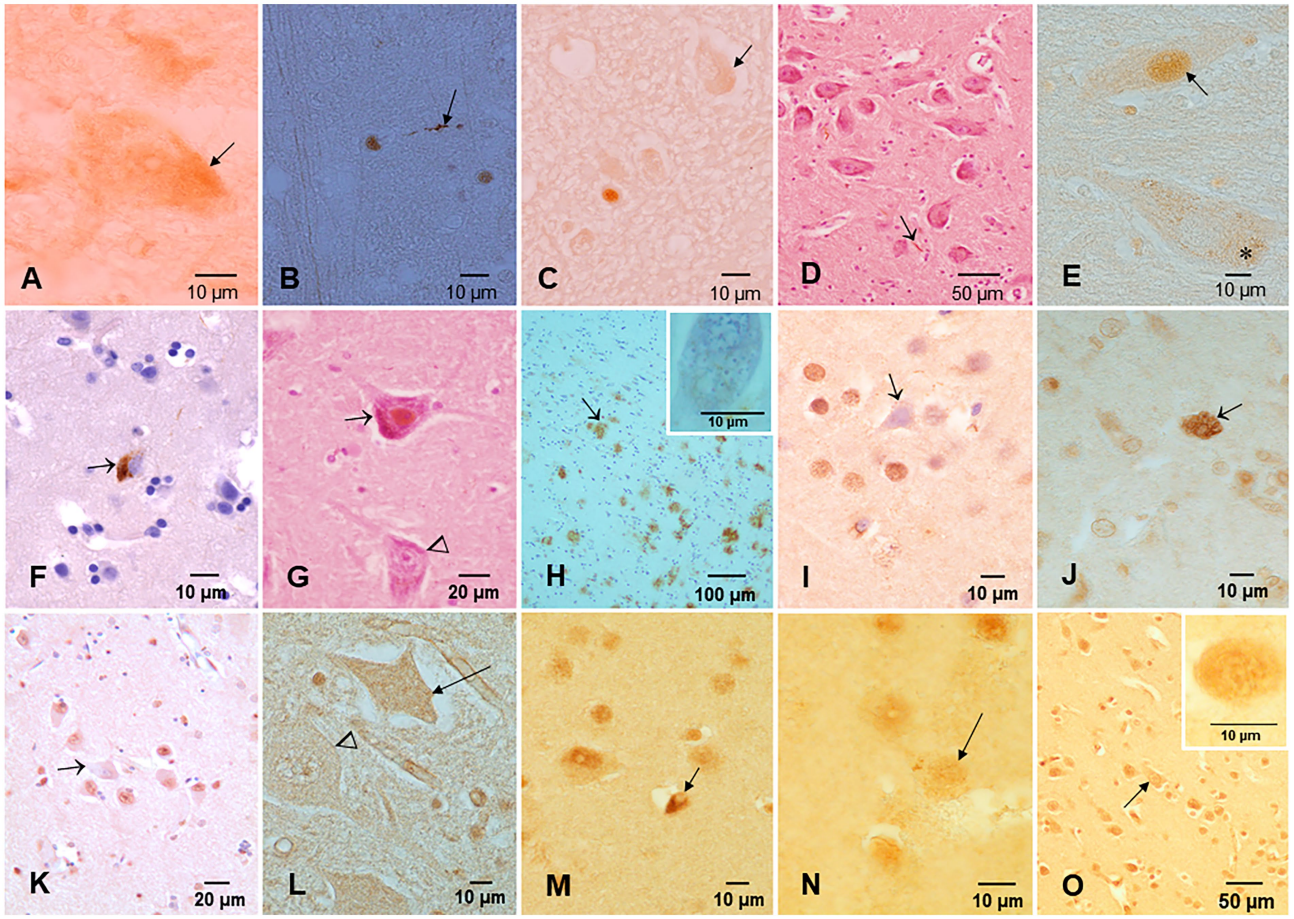
Protein aberrant pathology in MMC children and teens immunohistochemistry IHC. **(A)** Neuronal cytoplasmic tau (arrow) in substantia nigra of a 2-year old boy. **(B)** Tau positive neurite (arrow) in an 11-month-old baby brainstem. **(C)** Three-year-old boy with intracytoplasmic tau (arrow) and one positive tau nucleus, indicative of DNA damage. **(D)** Seventeen-year-old boy with positive P-tau neurites (arrow) in substantia nigra. **(E)** Same child as D with P-tau in both cytoplasm (*) and nuclear (arrow) locations. **(F)** Twenty-year-old male with frontal P-tau neuron (arrow). **(G)** Thirteen-year-old female with nuclear P-tau (arrow) in contrast with a negative neuron (arrow head). **(H)** Eleven-year-old boy temporal cortex with numerous AB42 plaques (arrow). The insert is a neuron with Hirano bodies in the same child. **(I)** TDP-43 IHC in the temporal cortex of a 20-year-old with a negative nucleus (arrow), in contrast with normal positive nuclei around the pyramidal neuron. **(J)** Six-month-old baby TDP-43 caudate showing a neuron with strong cytoplasmic positivity (arrow), surrounded by negative nuclei in neurons. **(K)** TDP-43 negative IR nucleus in hippocampal neuron (arrow) surrounded by positive neurons. **(L)** Six-month-old baby cervical motor neurons, TDP-43, show intracytoplasmic positivity (arrow), and the adjacent motor neuron shows negative nuclei (arrow head). **(M)** Fourteen-year-old female, temporal cortex IHC for TDP-43, an oligodendroglia shows a coiled positive tangle (arrow) with a negative nucleus. **(N)** Thirteen-year-old female temporal cortex TDP-43 IHC neuron (arrow) with positive granular staining. **(O)** Three-year-old boy temporal cortex TDP-43 IHC nucleus negative neuron (arrow). Insert shows a convoluted temporal neuronal nucleus with faint positive staining.

All 186 subjects showed P-tau in the brainstem, while diffuse amyloid cortical plaques and abnormal neurovascular units characterized young children (Figure 4). Children and young adults ≤20 years of age (n: 57) showed progressively more P-tau brainstem involvement, along with cortical tangles and amyloid plaques. Nuclear P-tau was identified in neurons, glia, and endothelium throughout the brain in the first two decades of life.

#### 3.2.1 Substantia nigra, locus coeruleus, olfactory bulb, and cerebellar neuropathology

**Figure 5:**
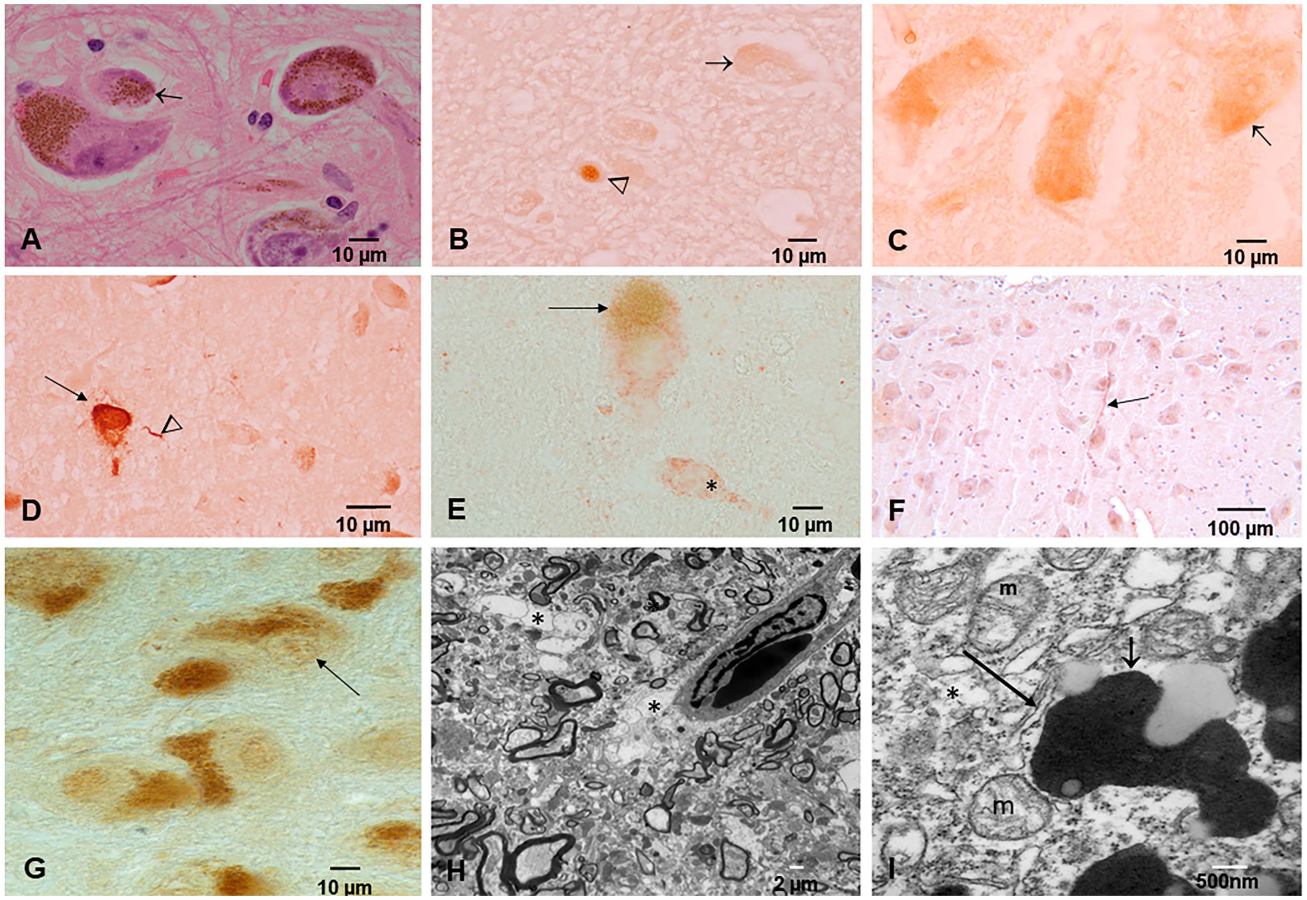
Substantia nigra (SN) neuropathology. **(A)** Macrophages packed with neuromelanin (arrow). HE staining. **(B)** P-tau nuclear (arrowhead) and cytoplasmic (arrow) in a 3-year-old. **(C)** Two-year-old neurons with abundant P-tau intracytoplasmic (arrow). **(D)** Forty-year-old male with neuronal P-tau tangles (arrow) and numerous P-tau positive neurites (arrowhead). **(E)** Thirty-two-year-old female neurons IHC for alpha-synuclein (red product). The * neuron exhibits red cytoplasmic α-Syn but very little neuromelanin pigment. **(F)** Thirteen-year-old female IHC for TDP-43 with a positive neurite (arrow). **(G)** Forty-four-year-old male TDp-43 IHC negative nuclear staining. **(H)** SN electron microscopy ×5,000; the perivascular neuropil is fragmented and vacuolated (*) with damage to the neurovascular unit. **(I)** SN EM ×50,000; neuromelanin (short arrow) with abundant NPs is seen surrounded by dilated endoplasmic reticulum (long arrow) and abnormal mitochondria (m).

The substantia nigra showed significant pathology at all ages, including positive P-tau neurites, numerous macrophages with neuromelanin, positive alpha-synuclein, and TDP-43 pathology (Figure 5). The neurovascular unit was damaged from infancy.

**Figure 6:**
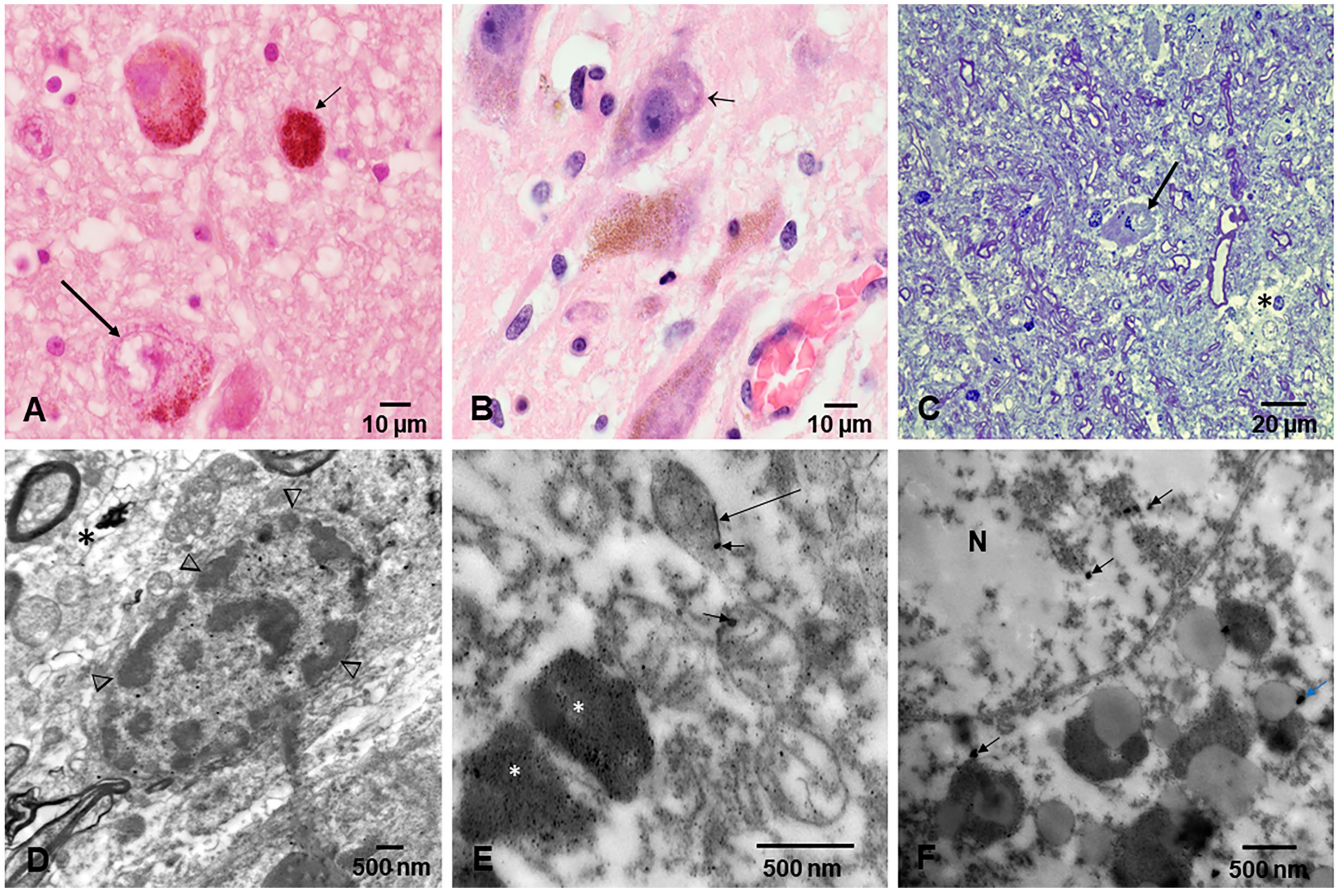
Locus coeruleus. **(A)** Thirteen-year-old female, the long arrow points to a partially degranulated neuron, while the small arrow shows a macrophage packed with neuromelanin close to a neuron. **(B)** Forty-year-old male with partially degranulated neurons (arrow) and surrounding macrophages. **(C)** Electron micrograph, Toluidine blue staining showing a neuron (arrow), and vacuolated, fragmented neuropil (*). **(D)** EM of a neuron with clumps of heterochromatin across the nuclear membrane (arrowheads) and surrounded by loose neuropil (*). **(E)** EM ×25,000 showing neuromelanin (*) containing numerous NPs, while mitochondria are abnormal and exhibit spherical NPs (short arrow) or nanorods (long arrow). Some of these nanorods contain titanium. **(F)** EM ×83,300 an abnormal nuclear matrix (N) containing NPs (short arrows) and numerous lysosomal bodies with NPs.

Younger than 10-year-old children exhibited pre-tangle stages a-c, 1a, and 1b, while those older than 11 years showed neurofibrillary NFTs stages I–V. P-tau location in the lower medulla sections included the reticular formation (lateral, medial, and raphe nucleus), dorsal motor neuron of the vagus (X), and spinal trigeminal nerve (V). The location of P-tau neurites, cytoplasmic P-tau, and tangles in children and teens between 11 and 20 years of age included the following: the central and periaqueductal gray, medial longitudinal fasciculus, gigantocellular reticular nucleus, dorsal motor vagal and solitary nuclei, intermediate reticular zone, reticulotegmental nucleus of the pons, medial lemniscus, trigeminal-thalamic ventral tract, nucleus ambiguous, pars compacta of the substantia nigra, pedunculopontine nucleus, spinal trigeminal nucleus, locus coeruleus, inferior colliculus, dorsal cochlear, and vestibular nuclei (see Figures 5, 6).

### 3.3 Electron microscopy

**Figure 7:**
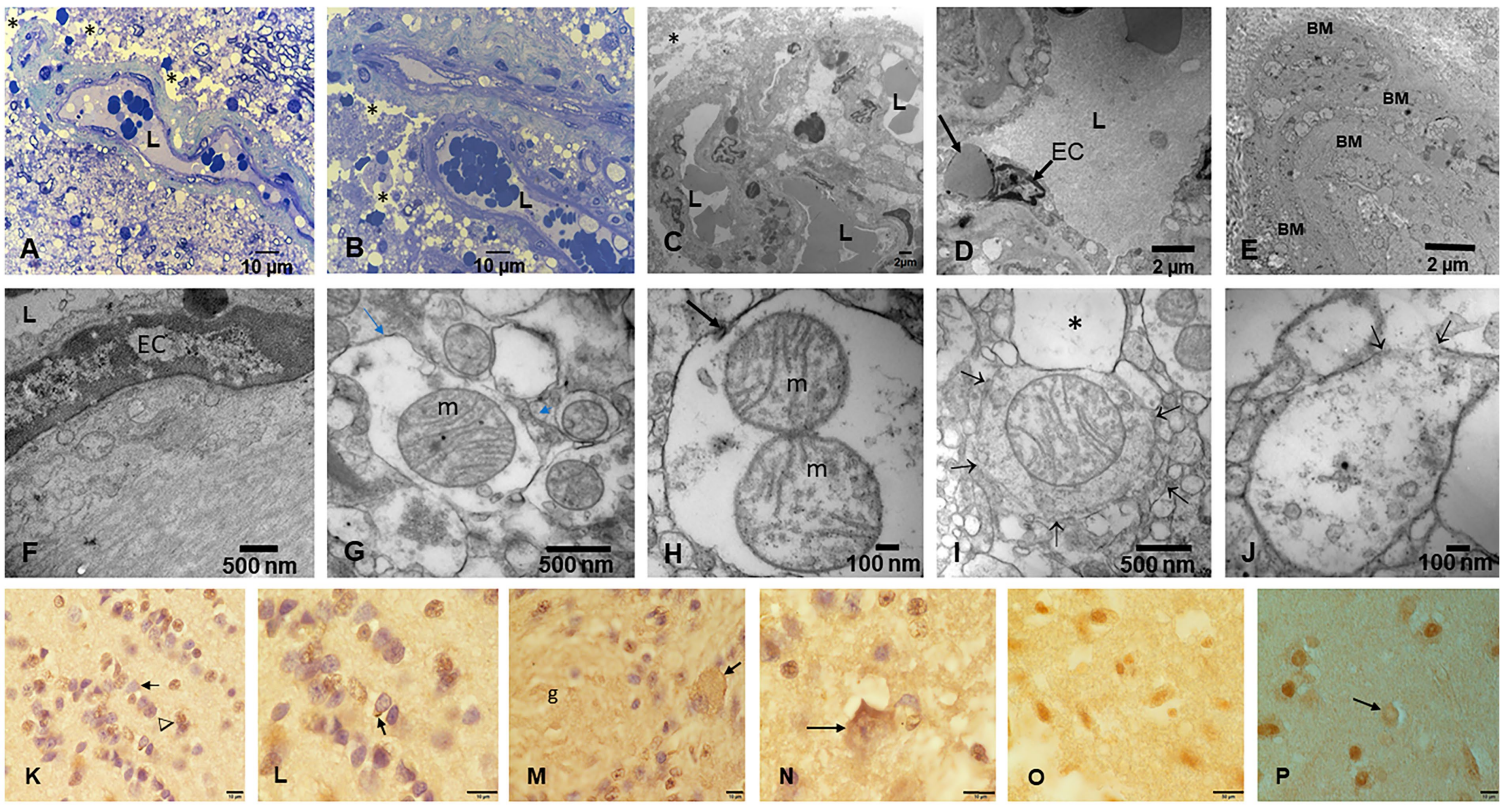
Olfactory bulb (OB) and entorhinal cortex. **(A)** Fourteen-year-old female, toluidine blue 1 μm showing an arteriolar vessel with thickening of the basement membranes. L is the vessels’ lumen and * shows the perivascular vacuolization and fragmentation of the neuropil. **(B)** Same child as A, the neurovascular unit damage (*) is extensive throughout the OB. **(C)** Electron micrograph ×3,000 OB in a 17-year-old male resident in a NE very polluted MMC area shows the abnormal basement membranes in this cluster of vessels. **(D)** Same child as in C, OB vessel showing a typical finding of both OB and cerebellum, the erythrophagocytosis of NPs loaded red blood cells (arrow) by ECs. **(E)** The onion bulb arrangement of OB vessel basement membranes is a very common finding in children and young adults. This is a 14-year-old girl resident in a polluted industrial MMC area. **(F)** Same 14-year-old with beta-amyloid sheets in OB, EM ×42,500. This child had extensive Aβ42 IHC positivity in the OB, frontal, and temporal cortex. **(G)** Twenty-four-year-old male OB, EM ×83,300, showing mitochondria with abnormal cristae and NPs surrounded by a double membrane lysosome-autophagy structure. **(H)** Same subject EM ×133,000 with two abnormal mitochondria (m) in a lysosome autophagic structure (arrow). Please notice the cellular debri inside the lysosome. **(I)** Fifteen-year-old-male OB, EM ×83,300, a lysosomal autophagic structure, note the adjacent empty double membrane structures (*). **(J)** Same 15-year-old as in I, OB, EM ×167,000, a large autophagic lysosome with debri and one combustion NP. The arrows show a breakdown of the double membrane. **(K)** Twenty-year-old male IHC OB, TDP-43 granule neurons-the most abundant inhibitory neurons-with negative nuclear staining (arrow), contrasting with positive ones (arrowhead). **(L)** OB Granule cells with positive TDP-43 tangles (arrow). **(M)** Glomerular OB region, TDP-43 IHC, same 20-year-old as K and L. GABAergic periglomerular interneurons with negative nuclei and positive staining against the plasma membrane (arrow). **(N)** TDP-43 positive cytoplasmic staining in a periglomerular neuron (arrow). **(O)** Thirty-nine-year-old male, anterior olfactory nucleus in the transition zone of the OB to the olfactory peduncle, IHC TDP-43, showing negative and positive neuronal nuclear staining. **(P)** Twenty-one-year-old male, TDP-43 IHC entorhinal cortex with one neuron showing cytoplasmic positive TDP-43 and a negative nucleus (arrow).

Electron microscopy changes were documented in every subject in the first and second decades, and strikingly, the olfactory bulb (OB) exhibited major neurovascular unit pathology with thickening and deposition of abnormal basement membrane (BM) layers (Figure 7). Vascular and neuropil beta pleated sheet helicoidal conformation fibers were common OB findings, lipofuscin was abundant, and NPs across endothelial BMs were common. NPs wih a range size of 8–66 nm in diameter were present in mitochondria (Figures 7G–I).

**Figure 8:**
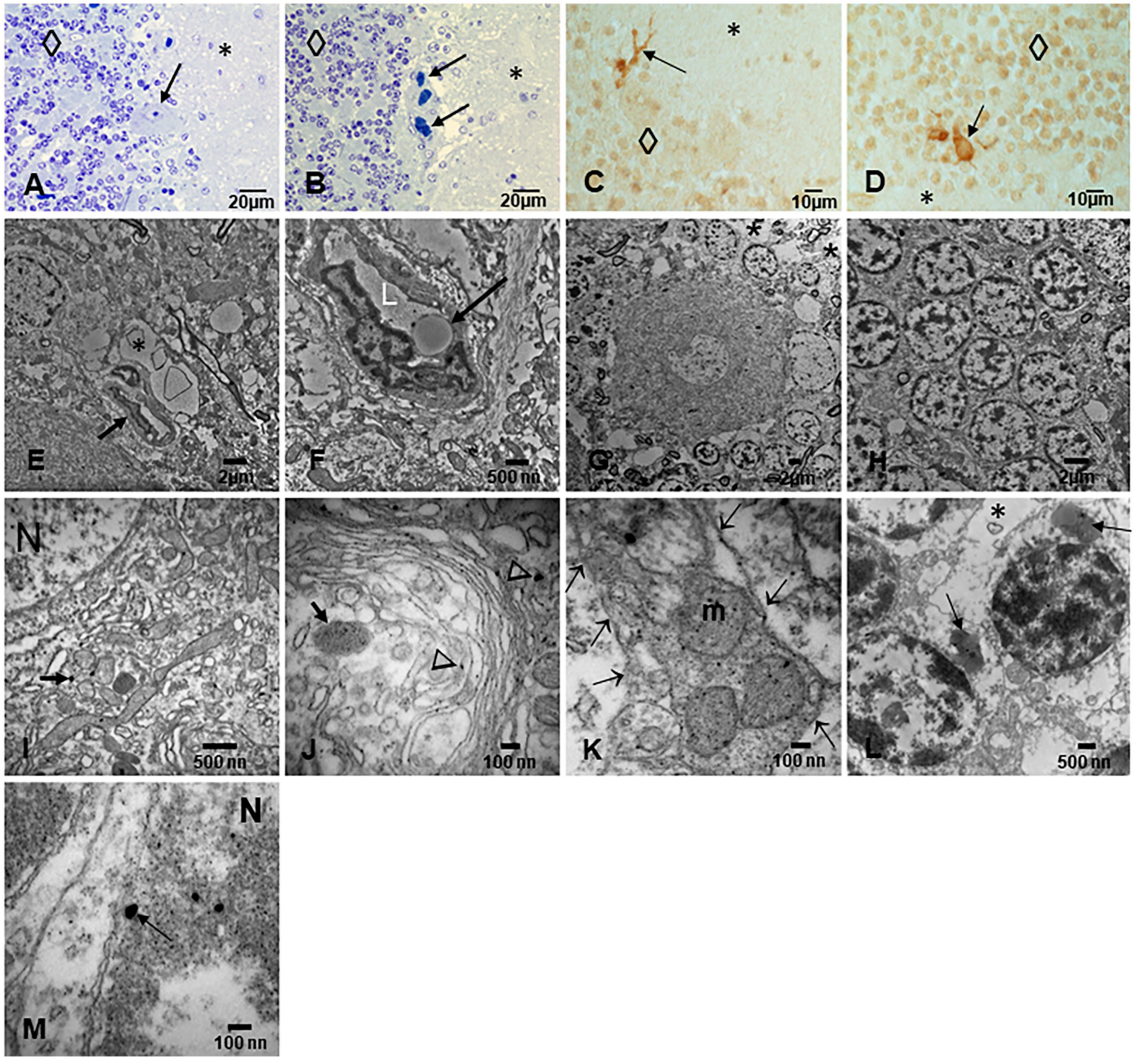
Cerebellum. **(A)** Toluidine blue 1 μm slide sections showing neocerebellum, left flocculonodular lobe, Purkinje cell (arrow), molecular layer (*), and granular layer (◊). **(B)** Six-month-old resident in MMC with a clustering of arteriolar blood vessels at the Purkinje cell layer (arrows), note the vacuolization of the perivascular spaces in the molecular layer (*). **(C)** Six-month-old baby, TDP-43 IHC lateral hemisphere, note the positive TDP-43 dendrite swellings (arrow) at the Purkinje cell layer. **(D)** In the same child, a TDP-43 positive tangle in a Golgi inhibitory interneuron (arrow), the nucleus is negative. **(E)** EM ×5,000 neo cerebellar cortex with a blood vessel surrounded by a vacuolated perivascular space (arrow) and multiple vesicular spaces in the neuropil (*). **(F)** EM ×15,000. RBC Erythrophagocytosis is shown in this EC (arrow). Note the extensive damage to the neurovascular unit. **(G)** EM ×3,000. Purkinje cell surrounded by granular cells and focal areas (*) of vacuolated neuropil. **(H)** EM ×5,000, granular cells with slight variation in the nuclear distribution of heterochromatin. **(I)** EM ×30,000. Purkinje cell nucleus at the upper left showing intranuclear NPs, also seen in mitochondria (arrow). **(J)** EM ×80,000. Purkinje cell cytoplasm with NPs in dilated endoplasmic reticulum (arrowheads), Golgi and in isolated lysosomes (arrow). **(K)** EM ×80,000 Cluster of abnormal mitochondria (m) with NPs surrounded by a double membrane fragmented (arrows) structure. **(L)** EM ×15,000 granular neurons containing lysosomes with numerous NPs (arrows). Note the * neuropil vacuolization around them. **(M)** EM ×50,000 the granular neuron nucleus with round NPs (arrow) in association with heterochromatin and a breakdown of the nuclear membrane.

The cerebellum (Figures 8A–M) was particularly involved by NVU pathology and strikingly by ECs erythrophagocytosis (Figure 8F), similar to the one described for the OB. The presence of NPs was significant in mitochondria, in relation to heterochromatin and in cytoplasmic lysosomal bodies (Figures 8I–M).

**Figure 9:**
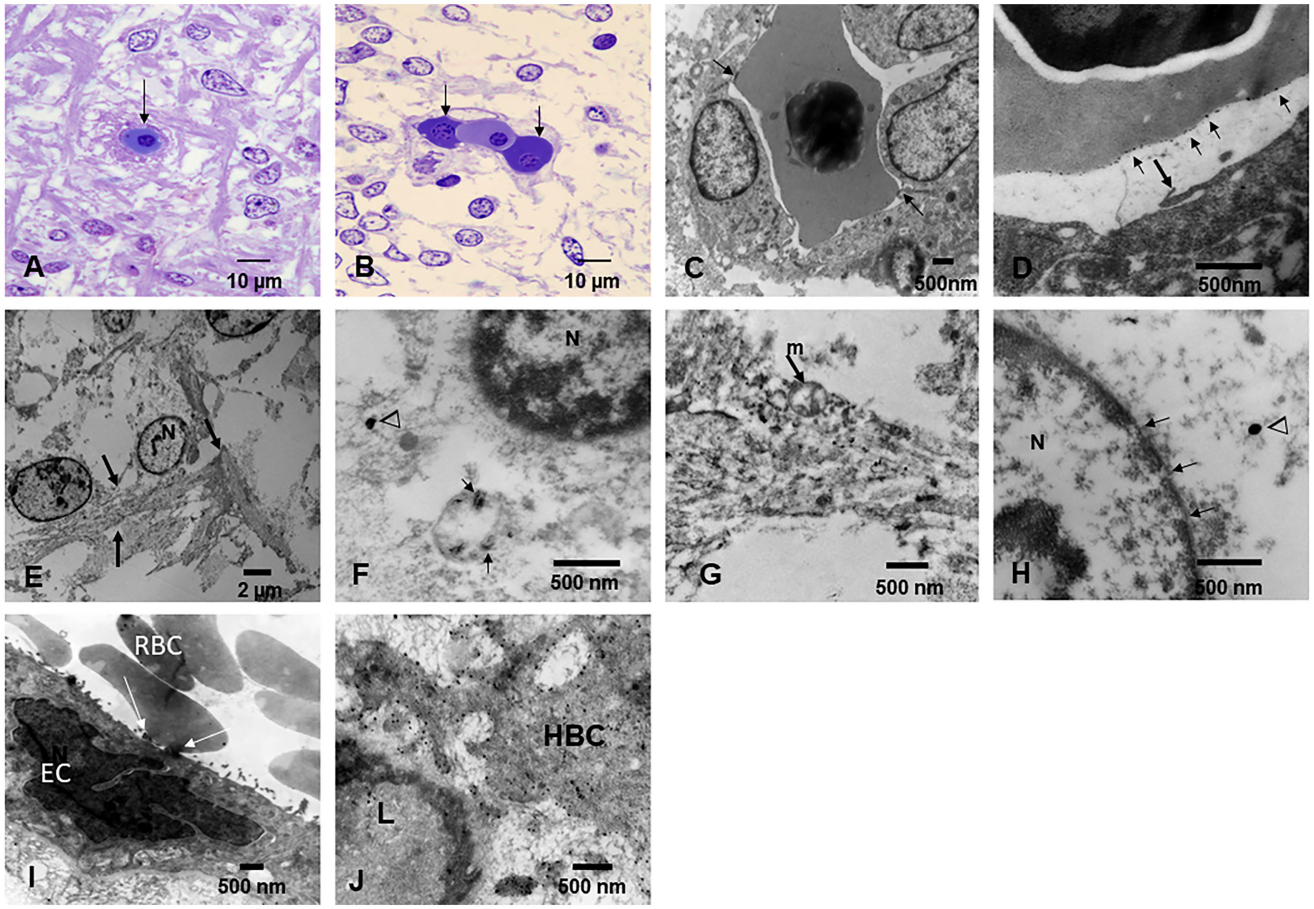
Fetal brain and placentas. **(A)** Fetal brain 12 weeks, Toluidine blue 1 μm sections. The central capillary contains an erythroblast—the ones that transport NPs. Note the fibers crossing in several directions in the primitive neuropil. **(B)** Fetal brain cortex with a capillary containing three erythroblasts in different stages of maturity. **(C)** EM ×25,000. A fetal brain capillary is occupied by an erythroblast. The arrows point to the contact surface between the erythroblast and the ECs, the place where the transfer of NPs takes place. **(D)** The fetal brain-activated ECs send filopodia (arrow) toward the erythroblast surface. **(E)** EM ×83,300 likely primitive glial fibers (arrows) crossing the loose neuropil. **(F)** EM ×50,000. A likely primitive neuron showing a small mitochondrion containing nanorod structures (arrows) and free in the cytoplasm spherical NPs (arrowheads) are identified. The nucleus is labeled N. **(G)** EM ×58,000. Cell processes of a likely radial glial cell containing numerous NPs inside mitochondria and intermingled with radial glial filaments. **(H)** EM ×83,300. Neuronal primitive cell with spherical NPs (arrowhead) and a nucleus (N) with fragmented nuclear membrane (arrows). **(I)** EM ×25,000. Fetal placenta with maternal RBC, transferring NPs at the point of contact (white arrows). Note the podocytes’ luminal ECs activity. **(J)** EM ×58,000 Hofbauer cell (HBC) cytoplasmic processes loaded with NPs are seen adjacent to a fetal placenta blood vessel (L lumen).

Fetal brains postnatal weeks 12–15 and their placentas (Figure 9) exhibited NPs in nuclei and cytoplasm from ECs, glial, and neuronal precursors (Figures 9F–I). The transfer of NPs from fetal erythroblasts was documented to fetal brain ECs (Figures 9C–E), while the maternal RBC transferred NPs to the fetal placental side (Figure 9J) and fetal Hofbauer cells were loaded with NPs (Figure 9K).

### 3.4 EDX findings

**Figure 10:**
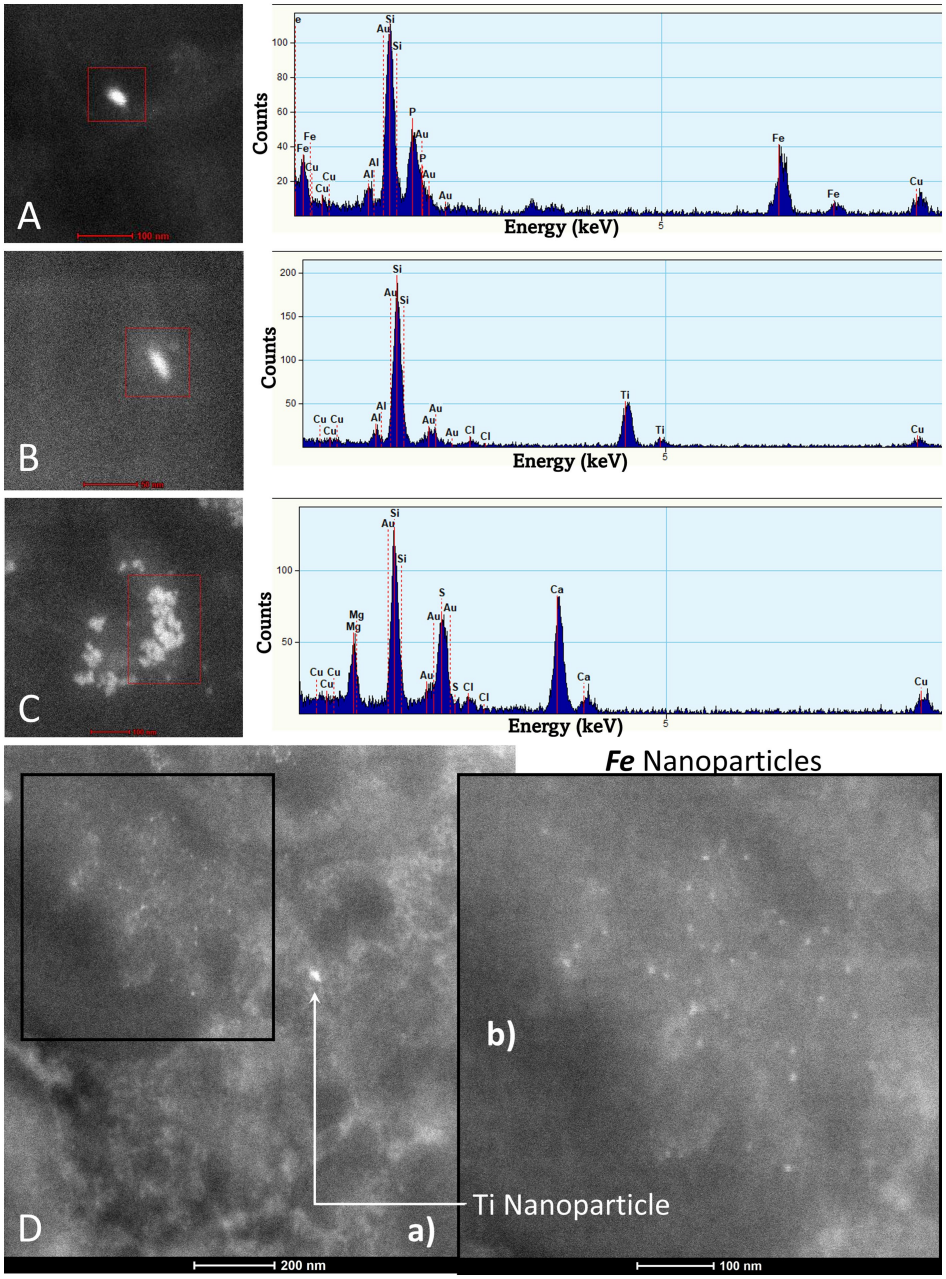
TEM Z-Contrast technique, NPs are documented in the caudate head from a 21-year-old male through EDX that shows the element’s peak. In **(A–C)**, it is confirmed that the presence of NPs composed of Fe, Ti, and Ca is common, while the other elements appearing in the spectra are part of the matrix embedding the tissue. Fe (10 nm) and Ti (25 nm) NPs are shown in **(D)**. The presence of Au and Cu is due to the materials from the Au grids.

EDX in individuals ≤20 years exhibited a wide spectrum of metals, metalloids, and non-metal elements, including Cesium (Ce), W (tin), Ti, Ni, Hg, Fe, Cu, Zn, Si, Na, P, and C. Figure 10 show the metal and metalloid profiles in individual UFPM/NPs in caudate neural and vascular cells analyzed by EDX.

**Figure 11:**
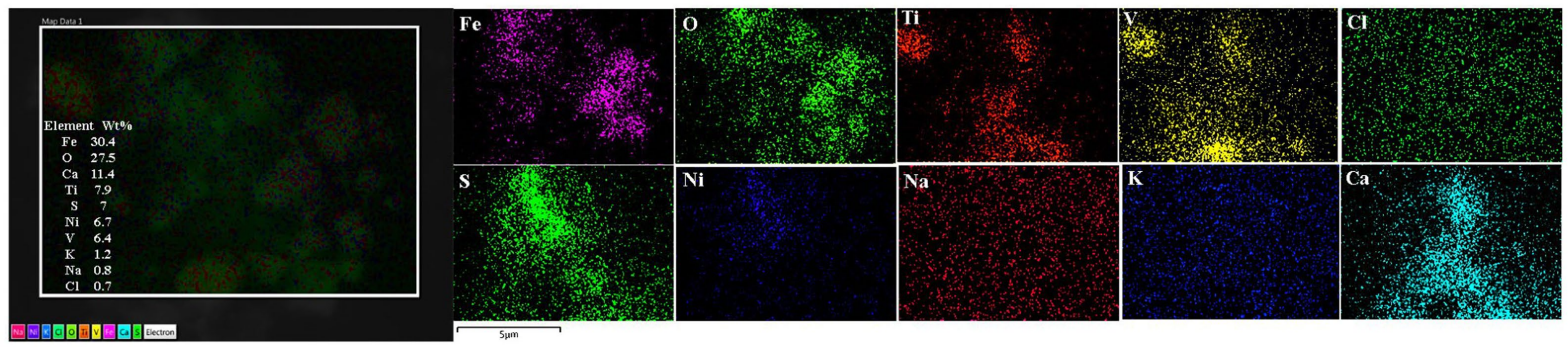
Olfactory bulb in a thirteen-year-old female resident in Northeast MMC, showing the common EDX spectrum of Fe, V, Ni, and S characteristic of industrial areas. Iron, oxygen, calcium, and titanium predominated in this sample. Chemical mapping showed the regions of Fe, O, Ti, V, and Ca are observed in the analyzed particle.

**Figure 12:**
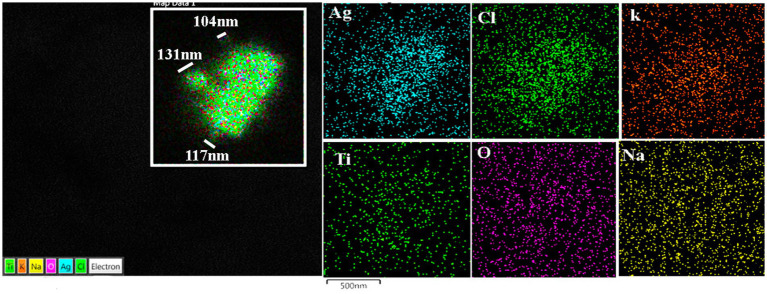
Prefrontal cortex in the same 13-year-old child as in Figure 11. Please notice the association of Ti with Ag in highly exposed NE children. MMC NE children have extensive brain frontal–parietal atrophy in MRI (please see Figure 14).

**Figure 13:**
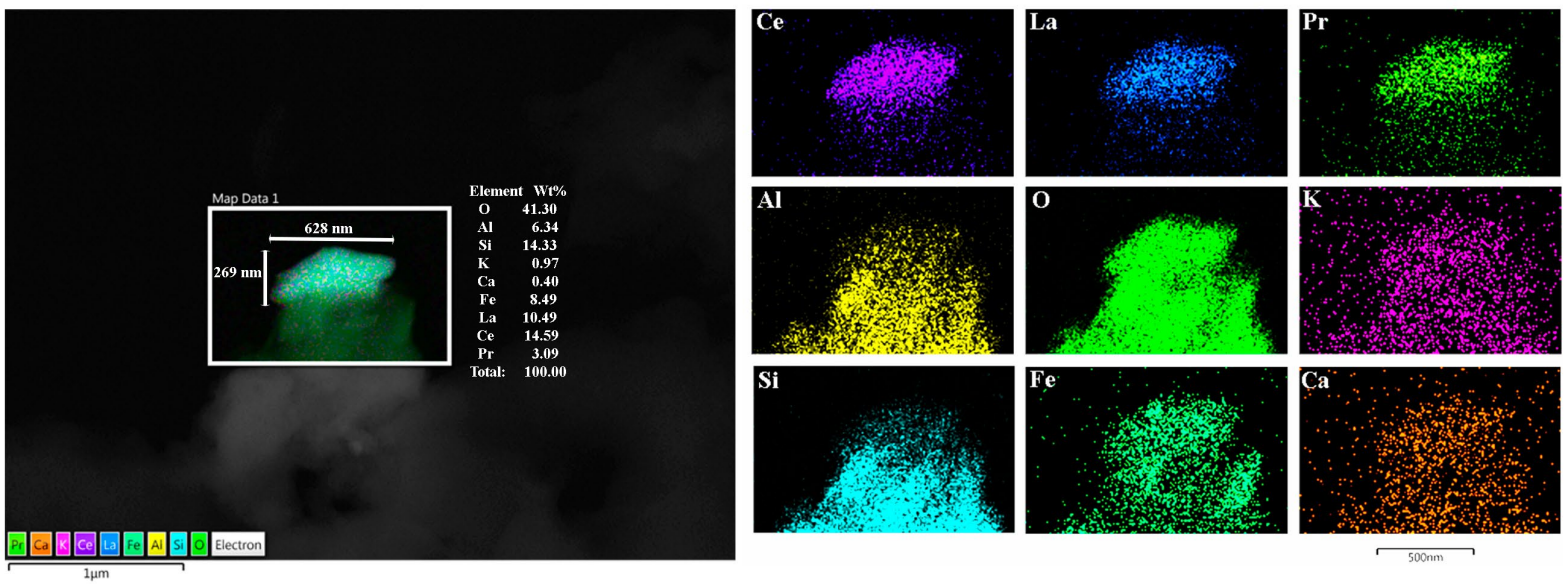
Frontal motor cortex Brodmann 4 in a 13-year boy, the presence of Lanthanum (La), Cerium (Ce), and Praseodymium (Pr) is associated with Fe, Al, and Si is striking. Ferrocerium is an alloy primarily composed of Fe, Ce, La, Nd, Pr, and Mg and is used widely in industry because of its pyrophoricity properties.

The spectrum of metal, metalloid, and natural NPs elements is illustrated in the OB and frontal cortex of MMC teens in Figures 11–13.

**Figure 14:**
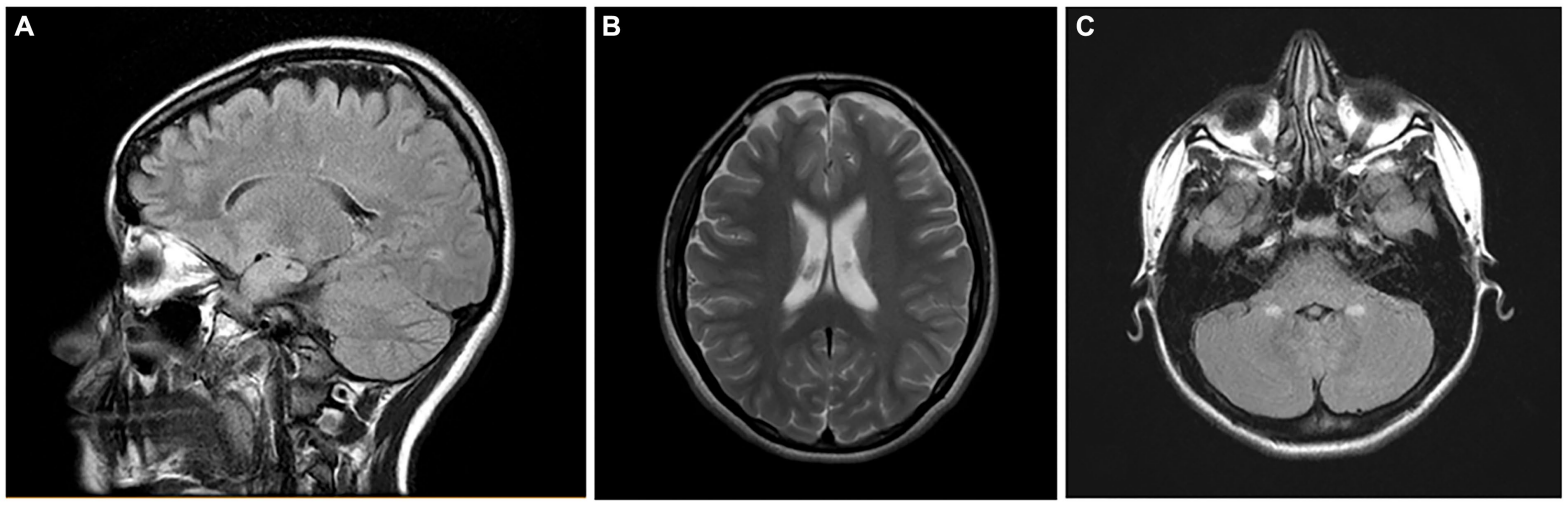
Brain MRIs in NE Mexico City children. **(A)** FLAIR sagittal brain image in a 14-year old shows the frontoparietal atrophy and white matter hyperintense lesions. **(B)** FLAIR coronal image in same child as **(A)** shows the ventricular enlargement and the bilateral frontoparietal atrophy. The child on **(C)** showed symmetrical cerebellar gliotic lesions associated with mild frontal parietal atrophy (not shown). Neurological findings included cerebellar gait and equilibrium alterations and cognitive impairment.

We are currently working on brain MRI volumetric studies in NE MMC children and young adults. We are documenting frontoparietal and cerebellar atrophy in teens residing less than 100 m from high-traffic heavy diesel vehicles (Figure 14).

## 4 Discussion

The key novel element of our research is the identification of the overlapping neuropathological hallmarks of fatal diseases, including AD, PD, FTLD, and ALS, in the first two decades of life in MMC residents. Twenty-two million people are being involuntarily exposed to high concentrations of PM_2.5_, including UFPM and NPs, throughout their lives, starting *in utero*. The complex atmospheric, occupational, and indoor lifelong exposures to highly neurotoxic pollutants complicate the neuropathological, nanotoxicological, cognitive, and neuropsychiatric clinical spectrum. Our findings are not isolated and ought to be examined in relationship with clinical, cognitive, brain volumetric MRIs and spectroscopies, sleep disturbances, brainstem auditory evoked potentials, olfactory alterations, and systemic and neural inflammation in MMC children and young adults (Calderón-Garcidueñas et al., 1995, 1996, 1997, 2008a,c, 2010, 2011, 2015a,b, 2019a,b, 2020b, 2022b).

All MMC children and young adults ≤20 years exhibited neuropathological hallmarks of fatal neurodegenerative diseases. The combination of AD + PD+/or TDP43 pathology is present in 14.8 ± 5.3 years children, which certainly obligates us to check carefully what type of emissions these young people were exposed to.

The complexity of the MMC atmosphere has been studied in the last three decades (Dunn et al., 2004; Kleinman et al., 2009; Múgica et al., 2010; Múgica-Alvarez et al., 2012; Kumar et al., 2014; Velasco and Retama, 2017; Molina et al., 2019; Velasco et al., 2019; Caudillo et al., 2020; Zavala et al., 2020; Gustin et al., 2023), and pollutants have changed during this period (Dunn et al., 2004; Múgica et al., 2010; Múgica-Alvarez et al., 2012; Morton-Bermea et al., 2014; Velasco et al., 2019). Platinum (Pt) has been increasing in MMC PM_2.5_ (Morton-Bermea et al., 2014), and the increment is associated with the abrasion and deterioration of automobile catalysts; about five million vehicles in MMC use catalytic converters.

The finding of lanthanum (La), cerium (Ce), and praseodymium (Pr) NPs is very interesting (Xu and Qu, 2014; Sadhu et al., 2016; Danmaliki and Saleh, 2017; Chen et al., 2019, 2023; He et al., 2019; Kumar et al., 2020; Morel et al., 2020; Guzman et al., 2022; Jubeer et al., 2023) given that these combinations of rare earth elements (REEs) are widely used in industry and are present in sunscreens, cosmetics, fiber optic cables, chemical and mechanical polishing, aircraft engines, welder’s and glass blower’s goggles, flints for lighters, and wood coating industry, and penetrate all biological barriers (Xu and Qu, 2014; Sadhu et al., 2016; Danmaliki and Saleh, 2017; Chen et al., 2019, 2023; He et al., 2019; Kumar et al., 2020; Morel et al., 2020; Guzman et al., 2022; Jubeer et al., 2023). Potentially very relevant to our findings of a combination of lanthanides and magnetic NPs is the fact that ZnFe_2_O_4_ nanotubes are a viable sorbent for dispersive micro-solid phase extraction of the trivalent ions of rare earth elements (Chen et al., 2019) and the combination of Pr with common Mn NPs gives rise to an incoherent spin reversal due to competition between the neighboring spins undergoing antiparallel to parallel spin rotations (Sadhu et al., 2016). The structure–property parallelism was cross-checked with the A-site vacant Pr0.9MnO3.2 NPs in the study by Sadhu et al. (2016) reflecting the crystal structure as a function of temperature. Lanthanides Ln^3+^ ions possess common and stable oxidation states except Ce^4+^, for which the f orbital is empty: 4f electron configurations determining magnetic moments, magnetic susceptibilities, and electronic relaxation time of Ln^3+^ions (Kumar et al., 2020) of great importance for magnetic NPs occupying key neural organelle, such as mitochondria.

The presence of lanthanides and heavy metal NPs, including Pt, Ti, Ag, Hg, Cd, Cr, Ni, and V, with a great variety of applications in material sciences, chemical industry, agriculture, biomedicine, coatings, phosphors, nanophosphors, and doped phosphorous NPs, plastics, nanofibers, nanowires, catalysts for automobile exhaust-gas treatments, oxidative coupling of methane and water-gas shift reaction and textiles, and as ignition elements in lighters and torches makes them a hazardous public health issue (Xu and Qu, 2014; Sadhu et al., 2016; Danmaliki and Saleh, 2017; Chen et al., 2019, 2023; He et al., 2019; Kumar et al., 2020; Morel et al., 2020; Guzman et al., 2022; Jubeer et al., 2023). The study by Morel et al. (2020) regarding CeNPs at the transcriptomic level, specifically affecting algal mobility, is very concerning for humans. Morel and collaborators emphasized the Ce NPs cell motility and organization impact in algae, supporting that bioavailability was different for ionic and particulate forms of Ce, and thus, extensive characterization is necessary to interpret complex exposures examining the biological effects of engineered nanomaterials. Transcriptomic studies are certainly needed to study neural cells exposed to NPs.

The presence of specific combustion, friction, and rare metals across the MMC and their seasonal variations are very relevant to the complex MMC quadruple aberrant pathology, and defining the brain NPs profiles according to residency within the 3,037 sq. mi will support the impact of industrial pollution, open-air trash, electronic waste, diesel NPs sources, as well as volcanic/agricultural and wildfire emissions from adjacent states and implement protective measures for highly exposed, marginalized MMC residents (McGuinn et al., 2020; Hurtado-Diaz et al., 2021; Servan-Mori et al., 2022; Wildfire Smoke Shrouds Mexico City, n.d.).

Given that the overlap of the neurodegenerative pathologies we see in MMC children are reported both in unimpaired and cognitively impaired older populations (Wakisaka et al., 2003; Kovacs et al., 2013; Boyle et al., 2018; Nag et al., 2018; Wennberg et al., 2019; Karanth et al., 2020; Robinson et al., 2023), early neurotoxic environmental factors must be identified, and preventive interventions executed in highly exposed populations. Moreover, early identification of pre-symptomatic stages, the definition of early progression and heterogeneity of the quadruple neurodegenerative hallmarks, and the contribution of brain structural changes to early neuropsychiatric symptoms need to be described (Benatar et al., 2023; Busy et al., 2023; Butler Pagnotti et al., 2023; Kapustin et al., 2023; Manca et al., 2023; Montero-Calle et al., 2023; Russell and Rohrer, 2023; Ulugut et al., 2023; Young et al., 2023).

The presence of NPs composed of metal, metalloid, and natural elements, associated with structural alterations of key organelles, is a major finding in brain and vascular cells in highly exposed MMC children (Calderón-Garcidueñas et al., 2008b, 2016, 2017, 2018, 2019c, 2022a,c). NPs are ubiquitous in the MMC atmosphere, omnipresent in public transport, and their composition coincides with key emission sources within the city (Dunn et al., 2004; Múgica et al., 2010; Múgica-Alvarez et al., 2012; Kumar et al., 2014; Calderón-Garcidueñas et al., 2019c; Velasco et al., 2019; Caudillo et al., 2020; Gustin et al., 2023). Moreover, NPs identified in the MMC atmosphere are also identified in brain tissues from 12- to 15-week-old fetuses and infants, teens, and young adults (Calderón-Garcidueñas et al., 2017, 2019c, 2022a) raising the question of their role in a number of pathological processes. These include the formation of aberrant overlapping proteins, the extensive structural subcellular nano neuropathology, CSF abnormalities, significant MRI-verified brain volumetric changes, systemic inflammation, and the multiple cognitive, auditory, olfactory, gait, and equilibrium disturbances documented in MMC young residents (Calderón-Garcidueñas et al., 2008c, 2015a, 2019a,b,c, 2020b, 2022a,b,c; González-Maciel et al., 2017).

The study by Hurtle et al. (2023) demonstrated that magnetic, metal, and metalloid NPs have the capacity to disrupt the highly coordinated process of liquid–liquid phase protein separation, thereby giving rise to unique and disease-specific biochemical protein signatures and abnormal protein depositions starting in childhood. As pointed out by Hurtle et al. (2023) examining biomolecular phase transitions will help our understanding of the molecular mechanisms mediating toxicity across diverse neurodegenerative diseases with NPs as a common denominator (Kovacs et al., 2013; González-Maciel et al., 2017; Calderón-Garcidueñas et al., 2018, 2019c; Wennberg et al., 2019; Karanth et al., 2020).

Redox-active, strongly magnetic, combustion, and friction-derived NPs are abundant in MMC air pollution, and AD continuum children and young MMC adults have higher numbers of brain NPs versus clean air controls (Calderón-Garcidueñas et al., 2019c). NPs surface charge, dynamic magnetic susceptibility, iron content, and redox activity contribute to ROS generation, NVU, mitochondria, and endoplasmic reticulum (ER) damage and are catalysts for protein misfolding, aggregation, and fibrillation (Calderón-Garcidueñas et al., 2019c). Although superparamagnetic iron oxide NPs (SPIONs) (Wolf et al., 2023) respond to external magnetic fields and are involved in cell damage by agglomeration/clustering, magnetic rotation and/or hyperthermia have been central to our cell damage knowledge in magnetic NPs, and the path mechanisms involving neural NPs damage are extensive (Hu et al., 2011; Mohammadipour et al., 2014; Jung et al., 2019; Li and Tang, 2023; Liu et al., 2023; Srikanth Vallabani et al., 2023; Tang et al., 2023; Wang et al., 2023). To compound the problem, since we are all continuously exposed to polymeric materials, nanoplastics capable of crossing the BBB and the GI barriers have to be included in the brain toxicity list (Kopatz et al., 2023; Zhao et al., 2023; Ziani et al., 2023). In addition, of deep interest for highly exposed subjects with damaged neurovascular units—in a similar fashion to metal NPs—the composition of the biomolecular corona surrounding the plastic nanoparticles is key for BBB passage, and cholesterol molecules enhanced their uptake (Kopatz et al., 2023; Zhao et al., 2023; Ziani et al., 2023).

It is becoming clear that MMC residents have targeted brain areas, as described in our brain MRI’s young adult results showing significant caudate and cerebellar atrophy (Calderón-Garcidueñas et al., 2022b) in keeping with their extensive electron microscopy pathology and the accumulation of magnetic NPs in these locations (Calderón-Garcidueñas et al., 2022a). Moreover, for MMC residents, we could link their residency with specific EDX profiles applicable to the highly industrialized and polluted NW and NE areas.

Given our brain MRI and clinical, cognitive, and forensic pathology results in MMC, it is urgent to have non-invasive biomarkers to identify children and young adults with AD/PD/TDP-43 early markers. Importantly, we should include clean air controls in our research studies. Such controls are missed in United States epidemiological brain MRI studies, mostly done in large cities with historically significant air pollution. Thus, current longitudinal brain volumetric studies in the USA and across the globe do not provide data that compare brain structures between residents in relatively non-polluted and significantly polluted environments (Frangou et al., 2022). Moreover, as toxicologists, we have a serious problem, given that the atmosphere varies from city to city (and many times within the same city), and a proxy such as PM_2.5_ is not telling us about the chemical gestalt to which we are exposed—including nanosized pollutants—thus, comparisons between cohorts from different cities, in different countries, are meaningless.

### 4.1 Summary

1. We need unbiased, well-trained, multidisciplinary groups, with access to state-of-the-art tools, instruments, and hospitals to do the needed research. Research in Mexico is constrained to an agenda that is not preventive, is not motivated to regulate heavy diesel vehicles, and has no viable health-accessible infrastructure.
2. Exposures to high concentrations of PM_2.5_ and UFPM/NPs likely play a significant role from intrauterine life onwards in the development of neurodegenerative processes. Early identification of pre-symptomatic stages, the definition of early progression and heterogeneity of the quadruple neurodegenerative hallmarks, and the contribution of brain structural changes to early neuropsychiatric symptoms must be made in MMC residents. The need for non-invasive biomarkers to identify children and young adults with AD/PD/TDP-43 early markers is urgent.
3. There is an overlap of AD, PD, and TDP-43 pathology in highly exposed MMC children and young adults, and the overlap is similar to the one shown in USA elderly subjects five to seven decades later (Kovacs et al., 2013; Wennberg et al., 2019; Karanth et al., 2020; Robinson et al., 2023). Moreover, AD hallmarks are present in 100% of the MMC forensic autopsy population starting at age 6 months to 40 years, which brings two critical issues to the discussion: 1. What is the pollutant exposure threshold for neurodegeneration? and 2. Since these young people did not have extra-neural gross and light microscopy pathology, comorbidities are unlikely to play a role in their brain pathology.

Equally critical is the fact that TDP-43 pathology was the second most frequent finding in young MMC residents. Thus, we should think of FTLD in the setting of cognitive impairment in MMC subjects, along with AD. It is remarkable that ALS is a rare disease in Mexico (Olivares et al., 1972; Martínez et al., 2011, 2020; Brown et al., 2021; López-Hernández et al., 2021) with a small series (Martínez et al., 2011) of Mexican patients (n:61), mostly male (1.8: 1 M:F), with a young mean age at the onset of 47.5 ± 10.5 years and a median interval from onset to diagnosis of 12 months. Spinal onset occurred in 66% of patients and an overall mean survival from onset of 68.6 months. Brown et al. (2021) estimated ALS prevalence and incidence in 22 countries across Europe, North America (United States and Canada), Latin America (Argentina, Brazil, Colombia, Mexico, and Uruguay), and Asia. The pooled prevalence rates and incidence rates were 6.22 and 2.31 for Europe, 5.20 and 2.35 for North America, and 3.41 and 1.25 for Latin America, respectively. In Mexico, ALS molecular genetics studies are scarce (Cervantes-Aragón et al., 2019), and there is also a failure to diagnose FTLD, so efforts are on the way to do proper diagnosis (Ibanez et al., 2021).

1. The relationship between air pollution, academic achievement, sleep disorders, neurodegeneration, depression, and suicide (ENLACE, 2013; Calderón-Garcidueñas et al., 2018, 2019b, 2020a, 2022a,b,c, 2023a,b; Gutiérrez-Pulido et al., 2020; Casas-Muñoz et al., 2023; García-Dolores et al., 2023; OECD, 2023a,b,c) ought to be a study focused on highly polluted urban centers. It is clear that academic achievement is low for Mexican children. For example, in 2006, 78.7% of elementary school children performed poorly in reading and 82.4% in mathematics (ENLACE, 2013).

In 2023, the Organization for Economic Cooperation and Development (OECD)—a forum of 41 democracies with market-based economies collaborating to develop policy standards to promote sustainable economic growth (OECD, 2023a,b,c) reported Mexican students’ mathematical performance for Program for International Student Assessment (PISA) (mathematical literacy of a 15-year-old to formulate, employ, and interpret mathematics in a variety of contexts to describe, predict, and explain phenomena), and reading performance (the capacity to understand, use, and reflect on written texts to achieve goals, develop knowledge and potential, and participate in society) ranked 37 out of 41 countries (OECD, 2023a,b).

Mexico underperforms the average in income, jobs, education, health, environmental quality, social connections, safety, and life satisfaction (OECD, 2023c). Adolescents in MMC have a high prevalence of conduct problems, depression, obsessive-compulsive disorder, live sexual violence, physical violence, negligence, bullying, and a mental health diagnosis in a family member as predictors of mental health problems and suicidal behavior (Calderón-Garcidueñas et al., 2020a; Casas-Muñoz et al., 2023; García-Dolores et al., 2023). For children and teens residing in MMC, there is an ongoing compromised, long-term educational and employment outcome into adulthood. As we put forward in our 2020 publication (Calderón-Garcidueñas et al., 2020a), the in-progress neurodegeneration in young MMC urbanites associated with suicide before COVID (Calderón-Garcidueñas et al., 2018) likely is playing a role in the increased suicide rates during COVID.

1. Quadruple abnormal neural proteins starting in infants and progressing as the subjects remain in the polluted environment should be concerning for health authorities as it has economic, academic, judicial, and social impacts for millions of people residing in such places. The health crisis in MMC is serious, and the cost–benefit ratio is in favor of acting. We need to protect public health from deadly UFPM and industrial NPs. We need accurate UFPM and NPs measurements, precise instruments, trained researchers, and guidelines to select the pollutants that we ought to be measuring. The problem of human exposure to particle pollution is solvable. We are knowledgeable about the main emission sources and the technological options to control them.

What are we waiting for?

## Data availability statement

The datasets for this study can be found in Supplemental Table 1. Further enquiries can be directed to the Corresponding Author.

## Ethics statement

The studies involving human participants were reviewed and approved by the University of Montana, Missoula, IRB# 206R-09 and IRB#185-20 for the Protection of Human Subjects in Research. Written informed consent to participate in the clinical study was provided by the participants/participants legal guardians.

## Author contributions

LC-G: Conceptualization, Data curation, Formal analysis, Funding acquisition, Investigation, Methodology, Project administration, Resources, Supervision, Validation, Visualization, Writing – original draft, Writing – review & editing. ES: Visualization, Writing – review & editing. RT-J: Air pollutant analysis, Investigation, Formal analysis, Writing – review & editing. JH-L: Conceptualization, Formal analysis, Investigation, Resources, Writing – review & editing. MA-M: Formal analysis, Investigation, Methodology, Resources, Writing – review & editing. AG-M: Data curation, Formal analysis, Resources, Supervision, Validation, Writing – review & editing. RR-R: Data curation, Formal analysis, Investigation, Resources, Writing – review & editing. BP-G: Conceptualization, Formal analysis, Resources, Supervision, Writing – review & editing. HS-P: Data curation, Formal analysis, Investigation, Methodology, Resources, Writing – review & editing. ST-C: Data curation, Formal analysis, Investigation, Methodology, Resources, Writing – review & editing. AR-G: Conceptualization, Investigation, Methodology, Resources, Writing – review & editing. IL: Conceptualization, Investigation, Resources, Writing – review & editing. CG-M: Formal analysis, Investigation, Resources, Writing – review & editing. RD: Conceptualization, Formal analysis, Investigation, Methodology, Resources, Writing – review & editing. AR: Statistical analysis. PM: Statistical analysis, Formal analysis, Supervision, Writing – review & editing.

## Footnotes

^1^http://www.r-project.org/
