# Supplementary material for: Alzheimer and Parkinson diseases, frontotemporal lobar degeneration and amyotrophic lateral sclerosis overlapping neuropathology start in the first two decades of life in pollution exposed urbanites and brain ultrafine particulate matter and industrial nanoparticles, including Fe, Ti, Al, V, Ni, Hg, Co, Cu, Zn, Ag, Pt, Ce, La, Pr and W are key players. Metropolitan Mexico City health crisis is in progress
Source: Front Hum Neurosci. 2024 Jan 12;17:1297467. doi: 10.3389/fnhum.2023.1297467 (PMC10811680; doi:10.3389/fnhum.2023.1297467)
Supplement: Supplementary file 1 [file Table_1.DOCX]

Supplementary Material

***Supplemental Table 1*:** Autopsy and neuropathological data for the 186 cases examined with H&E, PHF-tau8 phosphorylated at Ser199-202-Thr205, α-synuclein phosphorylated at Ser-129, LB509 and TDP-43 mab2G10 and rabbit polyclonal Ab recognizing N-terminal TDP-43.

| ID# | AGE | GENDER | APOE | AD pτ | AD Aβ | Brainstem pτ | Brainstem αSyn | Brainstem  TDP-43* | TDP-43 cortical§ |  |  |  |
| --- | --- | --- | --- | --- | --- | --- | --- | --- | --- | --- | --- | --- |
| 1 | 1 | 1 | 0 | 1 | 2 | 1 | 0 | 1 | 1 |  |  |  |
| 2 | 1.4 | 0 | 0 | 2 | 0 | 0 | 0 | 0 | 0 |  |  |  |
| 3 | 2 | 1 | 0 | 1 | 0 | 1 | 0 | 0 | 0 |  |  |  |
| 4 | 3 | 1 | 0 | 2 | 0 | 1 | 0 | 0 | 0 |  |  |  |
| 5 | 4 | 1 | 0 | 2 | 0 | 0 | 0 | 0 | 1 |  |  |  |
| 6 | 7 | 1 | 0 | 2 | 0 | 0 | 0 | 0 | 1 |  |  |  |
| 7 | 11 | 1 | 0 | 2 | 2 | 1 | 0 | 0 | 1 |  |  |  |
| 8 | 11 | 1 | 0 | 2 | 0 | 0 | 0 | 0 | 0 |  |  |  |
| 9 | 11 | 0 | 0 | 1 | 2 | 1 | 1 | 1 | 1 |  |  |  |
| 10 | 12 | 1 | 0 | 4 | 4 | 1 | 0 | 0 | 0 |  |  |  |
| 11 | 12 | 1 | 0 | 2 | 2 | 0 | 0 | 0 | 0 |  |  |  |
| 12 | 13 | 0 | 1 | 2 | 2 | 0 | 0 | 0 | 1 |  |  |  |
| 13 | 13 | 0 | 0 | 2 | 2 | 1 | 1 | 0 | 0 |  |  |  |
| 14 | 14 | 0 | 0 | 2 | 2 | 0 | 1 | 1 | 0 |  |  |  |
| 15 | 14 | 0 | 0 | 2 | 0 | 0 | 0 | 0 | 1 |  |  |  |
| 16 | 14 | 1 | 0 | 2 | 2 | 0 | 0 | 0 | 0 |  |  |  |
| 17 | 14 | 1 | 0 | 2 | 2 | 1 | 0 | 0 | 0 |  |  |  |
| 18 | 14 | 1 | 0 | 2 | 2 | 1 | 0 | 1 | 1 |  |  |  |
| 19 | 15 | 1 | 1 | 2 | 2 | 1 | 0 | 0 | 1 |  |  |  |
| 20 | 15 | 1 | 0 | 2 | 2 | 0 | 0 | 1 | 0 |  |  |  |
| 21 | 15 | 1 | 0 | 3 | 2 | 1 | 0 | 0 | 1 |  |  |  |
| 22 | 15 | 1 | 0 | 2 | 2 | 0 | 0 | 0 | 0 |  |  |  |
| 23 | 15 | 1 | 0 | 2 | 2 | 1 | 0 | 0 | 1 |  |  |  |
| 24 | 16 | 0 | 0 | 2 | 2 | 0 | 1 | 0 | 1 |  |  |  |
| 25 | 16 | 1 | 0 | 2 | 2 | 0 | 0 | 0 | 0 |  |  |  |
| 26 | 16 | 1 | 0 | 2 | 2 | 1 | 0 | 1 | 0 |  |  |  |
| 27 | 17 | 1 | 0 | 3 | 2 | 1 | 0 | 1 | 1 |  |  |  |
| 28 | 17 | 1 | 0 | 2 | 2 | 1 | 0 | 0 | 1 |  |  |  |
| 29 | 17 | 1 | 0 | 2 | 2 | 0 | 0 | 0 | 0 |  |  |  |
| 30 | 17 | 1 | 0 | 2 | 2 | 1 | 0 | 1 | 1 |  |  |  |
| 31 | 17 | 1 | 0 | 2 | 2 | 1 | 0 | 0 | 0 |  |  |  |
| 32 | 17 | 1 | 0 | 2 | 2 | 3 | 1 | 0 | 1 |  |  |  |
| 33 | 17 | 1 | 0 | 2 | 2 | 0 | 0 | 0 | 1 |  |  |  |
| 34 | 17 | 1 | 0 | 2 | 2 | 0 | 0 | 0 | 0 |  |  |  |
| 35 | 17 | 1 | 1 | 2 | 2 | 1 | 1 | 1 | 0 |  |  |  |
| 36 | 17 | 1 | 0 | 2 | 2 | 0 | 0 | 0 | 0 |  |  |  |
| 37 | 18 | 1 | 0 | 2 | 2 | 1 | 1 | 0 | 1 |  |  |  |
| 38 | 18 | 1 | 0 | 2 | 2 | 1 | 0 | 0 | 1 |  |  |  |
| 39 | 18 | 1 | 1 | 2 | 2 | 0 | 0 | 0 | 0 |  |  |  |
| 40 | 18 | 1 | 0 | 2 | 2 | 1 | 0 | 1 | 0 |  |  |  |
| 41 | 19 | 1 | 1 | 2 | 2 | 1 | 0 | 0 | 0 |  |  |  |
| 42 | 19 | 1 | 0 | 2 | 2 | 0 | 0 | 0 | 1 |  |  |  |
| 43 | 19 | 1 | 0 | 2 | 2 | 0 | 0 | 0 | 1 |  |  |  |
| 44 | 19 | 1 | 0 | 2 | 2 | 1 | 1 | 1 | 0 |  |  |  |
| 45 | 19 | 0 | 0 | 2 | 2 | 1 | 0 | 0 | 0 |  |  |  |
| 46 | 20 | 1 | 0 | 2 | 2 | 0 | 0 | 0 | 0 |  |  |  |
| 47 | 20 | 1 | 0 | 2 | 2 | 1 | 1 | 0 | 1 |  |  |  |
| 48 | 20 | 1 | 1 | 2 | 2 | 1 | 1 | 0 | 1 |  |  |  |
| 49 | 20 | 1 | 0 | 5 | 2 | 1 | 0 | 0 | 1 |  |  |  |
| 50 | 20 | 1 | 2 | 5 | 2 | 0 | 0 | 0 | 1 |  |  |  |
| 51 | 20 | 1 | 0 | 2 | 2 | 1 | 1 | 0 | 0 |  |  |  |
| 52 | 20 | 0 | 0 | 2 | 2 | 1 | 0 | 0 | 1 |  |  |  |
| 53 | 20 | 1 | 0 | 2 | 2 | 2 | 1 | 1 | 0 |  |  |  |
| 54 | 20 | 1 | 0 | 2 | 2 | 1 | 0 | 0 | 1 |  |  |  |
| 55 | 20 | 1 | 0 | 2 | 2 | 1 | 0 | 0 | 0 |  |  |  |
| 56 | 21 | 1 | 1 | 2 | 2 | 0 | 0 | 0 | 0 |  |  |  |
| 57 | 21 | 1 | 3 | 2 | 0 | 1 | 1 | 0 | 1 |  |  |  |
| 58 | 21 | 1 | 0 | 2 | 2 | 0 | 0 | 0 | 0 |  |  |  |
| 59 | 21 | 1 | 0 | 2 | 2 | 0 | 0 | 0 | 0 |  |  |  |
| 60 | 22 | 1 | 2 | 2 | 2 | 1 | 0 | 0 | 0 |  |  |  |
| 61 | 22 | 0 | 0 | 2 | 2 | 0 | 0 | 0 | 0 |  |  |  |
| 62 | 22 | 0 | 0 | 2 | 2 | 1 | 1 | 1 | 1 |  |  |  |
| 63 | 22 | 1 | 2 | 2 | 2 | 0 | 0 | 0 | 1 |  |  |  |
| 64 | 22 | 0 | 0 | 0 | 0 | 1 | 0 | 0 | 0 |  |  |  |
| 65 | 22 | 1 | 0 | 2 | 2 | 1 | 1 | 0 | 0 |  |  |  |
| 66 | 23 | 1 | 0 | 2 | 2 | 0 | 0 | 0 | 0 |  |  |  |
| 67 | 23 | 1 | 0 | 2 | 2 | 0 | 0 | 0 | 0 |  |  |  |
| 68 | 23 | 1 | 0 | 2 | 2 | 1 | 0 | 0 | 1 |  |  |  |
| 69 | 23 | 1 | 0 | 2 | 2 | 1 | 0 | 0 | 0 |  |  |  |
| 70 | 24 | 1 | 0 | 2 | 2 | 0 | 0 | 0 | 0 |  |  |  |
| 71 | 24 | 1 | 0 | 2 | 2 | 0 | 0 | 0 | 1 |  |  |  |
| 72 | 24 | 1 | 0 | 2 | 2 | 0 | 0 | 0 | 0 |  |  |  |
| 73 | 24 | 1 | 0 | 2 | 2 | 1 | 1 | 1 | 1 |  |  |  |
| 74 | 24 | 1 | 0 | 5 | 2 | 0 | 0 | 0 | 0 |  |  |  |
| 75 | 24 | 1 | 1 | 2 | 2 | 0 | 0 | 0 | 0 |  |  |  |
| 76 | 24 | 1 | 0 | 2 | 2 | 1 | 0 | 0 | 0 |  |  |  |
| 77 | 24 | 1 | 0 | 2 | 2 | 1 | 0 | 0 | 1 |  |  |  |
| 78 | 24 | 1 | 2 | 5 | 2 | 0 | 0 | 0 | 0 |  |  |  |
| 79 | 24 | 1 | 0 | 2 | 2 | 0 | 0 | 0 | 1 |  |  |  |
| 80 | 25 | 1 | 0 | 2 | 2 | 1 | 1 | 0 | 1 |  |  |  |
| 81 | 25 | 0 | 0 | 2 | 2 | 1 | 0 | 0 | 0 |  |  |  |
| 82 | 25 | 1 | 0 | 1 | 2 | 0 | 0 | 0 | 0 |  |  |  |
| 83 | 25 | 1 | 0 | 2 | 2 | 1 | 0 | 0 | 0 |  |  |  |
| 84 | 25 | 1 | 1 | 5 | 2 | 1 | 0 | 0 | 1 |  |  |  |
| 85 | 25 | 1 | 0 | 2 | 2 | 0 | 1 | 0 | 0 |  |  |  |
| 86 | 26 | 1 | 0 | 2 | 2 | 0 | 0 | 0 | 0 |  |  |  |
| 87 | 26 | 0 | 0 | 2 | 2 | 0 | 0 | 0 | 1 |  |  |  |
| 88 | 26 | 1 | 0 | 2 | 2 | 0 | 0 | 0 | 1 |  |  |  |
| 89 | 26 | 1 | 0 | 2 | 2 | 1 | 1 | 0 | 1 |  |  |  |
| 90 | 26 | 1 | 0 | 2 | 2 | 0 | 1 | 0 | 0 |  |  |  |
| 91 | 26 | 1 | 0 | 2 | 2 | 0 | 0 | 0 | 0 |  |  |  |
| 92 | 27 | 1 | 0 | 2 | 2 | 1 | 0 | 0 | 0 |  |  |  |
| 93 | 27 | 1 | 1 | 2 | 2 | 0 | 0 | 0 | 1 |  |  |  |
| 94 | 27 | 1 | 0 | 2 | 2 | 1 | 1 | 1 | 1 |  |  |  |
| 95 | 27 | 1 | 0 | 2 | 2 | 0 | 0 | 0 | 0 |  |  |  |
| 96 | 27 | 1 | 0 | 2 | 2 | 2 | 1 | 1 | 1 |  |  |  |
| 97 | 27 | 1 | 0 | 2 | 2 | 1 | 0 | 0 | 0 |  |  |  |
| 98 | 27 | 1 | 0 | 2 | 2 | 0 | 0 | 0 | 1 |  |  |  |
| 99 | 27 | 1 | 0 | 2 | 2 | 0 | 0 | 0 | 0 |  |  |  |
| 100 | 27 | 1 | 0 | 2 | 2 | 1 | 1 | 1 | 0 |  |  |  |
| 101 | 28 | 1 | 0 | 2 | 2 | 1 | 0 | 0 | 0 |  |  |  |
| 102 | 28 | 0 | 1 | 5 | 3 | 0 | 0 | 0 | 0 |  |  |  |
| 103 | 29 | 1 | 0 | 2 | 2 | 1 | 0 | 0 | 0 |  |  |  |
| 104 | 29 | 1 | 0 | 2 | 2 | 0 | 0 | 0 | 1 |  |  |  |
| 105 | 29 | 0 | 0 | 2 | 2 | 1 | 1 | 1 | 1 |  |  |  |
| 106 | 30 | 1 | 0 | 2 | 2 | 1 | 0 | 1 | 0 |  |  |  |
| 107 | 30 | 1 | 0 | 2 | 2 | 0 | 0 | 0 | 0 |  |  |  |
| 108 | 30 | 1 | 0 | 2 | 2 | 1 | 0 | 0 | 0 |  |  |  |
| 109 | 30 | 1 | 0 | 2 | 2 | 0 | 0 | 0 | 0 |  |  |  |
| 110 | 31 | 1 | 0 | 2 | 2 | 1 | 1 | 0 | 0 |  |  |  |
| 111 | 31 | 1 | 0 | 2 | 2 | 0 | 0 | 0 | 1 |  |  |  |
| 112 | 31 | 1 | 0 | 2 | 2 | 1 | 0 | 1 | 1 |  |  |  |
| 113 | 31 | 1 | 1 | 5 | 3 | 1 | 0 | 0 | 0 |  |  |  |
| 114 | 31 | 1 | 1 | 5 | 3 | 0 | 0 | 0 | 1 |  |  |  |
| 115 | 31 | 1 | 0 | 2 | 2 | 0 | 0 | 0 | 1 |  |  |  |
| 116 | 32 | 0 | 2 | 5 | 3 | 1 | 0 | 1 | 0 |  |  |  |
| 117 | 32 | 1 | 0 | 2 | 2 | 2 | 0 | 1 | 1 |  |  |  |
| 118 | 32 | 1 | 0 | 2 | 2 | 1 | 1 | 0 | 0 |  |  |  |
| 119 | 32 | 1 | 0 | 5 | 3 | 0 | 0 | 0 | 0 |  |  |  |
| 120 | 32 | 1 | 0 | 2 | 2 | 0 | 0 | 0 | 0 |  |  |  |
| 121 | 32 | 1 | 0 | 4 | 2 | 1 | 1 | 0 | 0 |  |  |  |
| 122 | 32 | 1 | 0 | 4 | 2 | 1 | 0 | 0 | 1 |  |  |  |
| 123 | 33 | 1 | 0 | 2 | 2 | 0 | 0 | 0 | 0 |  |  |  |
| 124 | 33 | 1 | 0 | 2 | 2 | 1 | 0 | 1 | 1 |  |  |  |
| 125 | 34 | 1 | 0 | 2 | 2 | 1 | 1 | 1 | 1 |  |  |  |
| 126 | 34 | 1 | 0 | 2 | 2 | 0 | 0 | 0 | 0 |  |  |  |
| 127 | 34 | 1 | 0 | 4 | 2 | 0 | 0 | 0 | 1 |  |  |  |
| 128 | 34 | 1 | 0 | 3 | 2 | 1 | 0 | 1 | 1 |  |  |  |
| 129 | 34 | 1 | 0 | 3 | 2 | 1 | 0 | 0 | 0 |  |  |  |
| 130 | 34 | 1 | 0 | 5 | 3 | 0 | 0 | 0 | 0 |  |  |  |
| 131 | 34 | 1 | 0 | 2 | 2 | 0 | 0 | 0 | 0 |  |  |  |
| 132 | 34 | 0 | 0 | 2 | 2 | 1 | 1 | 0 | 0 |  |  |  |
| 133 | 35 | 1 | 0 | 2 | 2 | 1 | 0 | 0 | 1 |  |  |  |
| 134 | 35 | 1 | 0 | 3 | 2 | 0 | 0 | 0 | 1 |  |  |  |
| 135 | 35 | 1 | 0 | 2 | 2 | 1 | 0 | 0 | 0 |  |  |  |
| 136 | 35 | 0 | 0 | 2 | 2 | 1 | 1 | 0 | 0 |  |  |  |
| 137 | 35 | 1 | 0 | 5 | 3 | 1 | 0 | 0 | 0 |  |  |  |
| 138 | 35 | 1 | 0 | 3 | 2 | 2 | 1 | 1 | 1 |  |  |  |
| 139 | 35 | 1 | 0 | 5 | 3 | 2 | 0 | 0 | 1 |  |  |  |
| 140 | 36 | 0 | 0 | 2 | 2 | 0 | 0 | 0 | 0 |  |  |  |
| 141 | 36 | 1 | 1 | 5 | 3 | 3 | 1 | 1 | 1 |  |  |  |
| 142 | 36 | 1 | 0 | 3 | 2 | 1 | 1 | 1 | 1 |  |  |  |
| 143 | 36 | 1 | 0 | 3 | 2 | 2 | 0 | 0 | 0 |  |  |  |
| 144 | 36 | 1 | 0 | 2 | 2 | 1 | 0 | 0 | 0 |  |  |  |
| 145 | 36 | 1 | 0 | 4 | 2 | 0 | 0 | 0 | 0 |  |  |  |
| 146 | 36 | 1 | 0 | 4 | 3 | 0 | 0 | 0 | 1 |  |  |  |
| 147 | 37 | 1 | 0 | 5 | 3 | 1 | 0 | 0 | 1 |  |  |  |
| 148 | 37 | 1 | 0 | 3 | 2 | 0 | 0 | 0 | 0 |  |  |  |
| 149 | 37 | 1 | 0 | 3 | 2 | 0 | 0 | 0 | 0 |  |  |  |
| 150 | 37 | 0 | 0 | 5 | 4 | 1 | 1 | 1 | 1 |  |  |  |
| 151 | 37 | 1 | 0 | 3 | 2 | 1 | 1 | 1 | 1 |  |  |  |
| 152 | 37 | 1 | 0 | 2 | 2 | 0 | 0 | 0 | 1 |  |  |  |
| 153 | 38 | 1 | 0 | 2 | 2 | 0 | 0 | 0 | 0 |  |  |  |
| 154 | 38 | 1 | 0 | 4 | 3 | 1 | 0 | 0 | 0 |  |  |  |
| 155 | 38 | 1 | 0 | 4 | 3 | 1 | 0 | 0 | 1 |  |  |  |
| 156 | 38 | 1 | 0 | 3 | 2 | 0 | 0 | 0 | 0 |  |  |  |
| 157 | 38 | 1 | 0 | 3 | 2 | 0 | 0 | 0 | 0 |  |  |  |
| 158 | 38 | 1 | 2 | 5 | 3 | 2 | 1 | 0 | 0 |  |  |  |
| 159 | 39 | 1 | 0 | 3 | 2 | 0 | 0 | 0 | 0 |  |  |  |
| 160 | 39 | 1 | 0 | 4 | 3 | 1 | 0 | 0 | 1 |  |  |  |
| 161 | 39 | 1 | 0 | 4 | 3 | 1 | 0 | 0 | 1 |  |  |  |
| 162 | 39 | 1 | 1 | 4 | 3 | 1 | 0 | 0 | 0 |  |  |  |
| 163 | 39 | 1 | 0 | 3 | 2 | 0 | 0 | 0 | 0 |  |  |  |
| 164 | 39 | 1 | 0 | 3 | 2 | 1 | 1 | 1 | 1 |  |  |  |
| 165 | 39 | 0 | 0 | 4 | 3 | 1 | 0 | 0 | 0 |  |  |  |
| 166 | 39 | 1 | 0 | 3 | 2 | 0 | 0 | 0 | 0 |  |  |  |
| 167 | 39 | 1 | 0 | 3 | 2 | 0 | 0 | 0 | 0 |  |  |  |
| 168 | 39 | 1 | 0 | 4 | 3 | 1 | 0 | 0 | 1 |  |  |  |
| 169 | 39 | 1 | 0 | 3 | 2 | 0 | 0 | 0 | 0 |  |  |  |
| 170 | 39 | 1 | 0 | 4 | 2 | 0 | 0 | 0 | 0 |  |  |  |
| 171 | 39 | 1 | 0 | 5 | 3 | 1 | 1 | 0 | 1 |  |  |  |
| 172 | 40 | 1 | 0 | 4 | 2 | 0 | 0 | 0 | 1 |  |  |  |
| 173 | 40 | 1 | 0 | 4 | 3 | 1 | 1 | 1 | 1 |  |  |  |
| 174 | 40 | 1 | 0 | 5 | 3 | 0 | 0 | 1 | 1 |  |  |  |
| 175 | 40 | 1 | 0 | 5 | 4 | 0 | 0 | 0 | 0 |  |  |  |
| 176 | 45 | 1 | 0 | 4 | 3 | 1 | 0 | 0 | 0 |  |  |  |
| 177 | 50 | 1 | 0 | 5 | 3 | 0 | 1 | 1 | 1 |  |  |  |
| 178 | 54 | 1 | 0 | 3 | 2 | 1 | 0 | 0 | 1 |  |  |  |
| 179 | 61 | 1 | 0 | 3 | 2 | 0 | 0 | 0 | 1 |  |  |  |
| 180 | 71 | 1 | 0 | 4 | 3 | 1 | 1 | 0 | 0 |  |  |  |
| 181 | 75 | 0 | 0 | 4 | 3 | 1 | 1 | 0 | 0 |  |  |  |
| 182 | 85 | 0 | 0 | 4 | 3 | 1 | 0 | 0 | 1 |  |  |  |

183 6m 1 0 1 1 1 1 1 1

184 13 1 0 3 2 1 0 1 1

185 21 1 1 3 2 1 1 1 1

186 77 1 1 4 4 1 1 1 1

Gender: 0=female, 1=male. APOE 0=3/3, 1=3/4, 2=4/4, 3=2/3

AD staging pτ Stage: 0=absent, 1= pre-tangle stages a-c, 2= pre-tangle stages 1a,1b, 3=NFT stages I, II, 4=NFT stages III-IV, 5=NFT stages V-VI

AD staging Aβ Phase: 0=absent, 1=basal temporal neocortex, 2=all cerebral cortex, 3=subcortical portions forebrain, 4=mesencephalic components, 5=Reticular formation and cerebellum.

Substantia nigrae pτ was evaluated as none=0, pre-tangles,positive neurites, and tangles using the PHF-tau8 phosphorylated at Ser199-202-Thr205 =1(Innogenetics, Belgium, AT-8 1:1000).

Substantia nigrae α-S was evaluated as none=0 and neuronal immunoreactive (IR) aggregates in the somato-dendritic compartment, cytoplasmic inclusions, core-halo Lewy bodies and dystrophic neurites (Lewy neurites)=1, using α-synuclein phosphorylated at Ser-129, LB509 (In Vitrogen, Carlsbad, CA 1:1000)

* Brainstem TDP-43 using two Ab was evaluated as none=0 and dash-like IR particles in the vicinity of the cell nucleus, with or without complete loss of nuclear TDP-43 expression and somatic skein-inclusions=1, using mab2G10 (Roboscreen GmbH, Leipzig, Germany 1:1000) and Proteintech TDP-43 rabbit polyclonal antibody recognizing the N-terminal TDP-43 (Proteintech #10782-2-AP).

§ TDP-43 in Frontal, temporal, hippocampal, cerebellar and cervical C1-C2 evaluated as none=0 and dash-like IR particles in the vicinity of the cell nucleus, with or without complete loss of nuclear TDP-43 expression and somatic skein-inclusions=1, using mab2G10 (Roboscreen GmbH, Leipzig, Germany 1:1000) and Proteintech TDP-43 rabbit polyclonal antibody recognizing the N-terminal TDP-43 (Proteintech #10782-2-AP).
